# Liver Necrosis and Lethal Systemic Inflammation in a Murine Model of *Rickettsia typhi* Infection: Role of Neutrophils, Macrophages and NK Cells

**DOI:** 10.1371/journal.pntd.0004935

**Published:** 2016-08-22

**Authors:** Stefanie Papp, Kristin Moderzynski, Jessica Rauch, Liza Heine, Svenja Kuehl, Ulricke Richardt, Heidelinde Mueller, Bernhard Fleischer, Anke Osterloh

**Affiliations:** 1 Department of Immunology, Bernhard Nocht Institute for Tropical Medicine, Hamburg, Germany; 2 Institute for Immunology, University Medical Center Hamburg-Eppendorf, Hamburg, Germany; University of Liverpool, UNITED KINGDOM

## Abstract

*Rickettsia* (*R*.) *typhi* is the causative agent of endemic typhus, an emerging febrile disease that is associated with complications such as pneumonia, encephalitis and liver dysfunction. To elucidate how innate immune mechanisms contribute to defense and pathology we here analyzed *R*. *typhi* infection of CB17 SCID mice that are congenic to BALB/c mice but lack adaptive immunity. CB17 SCID mice succumbed to *R*. *typhi* infection within 21 days and showed high bacterial load in spleen, brain, lung, and liver. Most evident pathological changes in *R*. *typhi*-infected CB17 SCID mice were massive liver necrosis and splenomegaly due to the disproportionate accumulation of neutrophils and macrophages (MΦ). Both neutrophils and MΦ infiltrated the liver and harbored *R*. *typhi*. Both cell populations expressed iNOS and produced reactive oxygen species (ROS) and, thus, exhibited an inflammatory and bactericidal phenotype. Surprisingly, depletion of neutrophils completely prevented liver necrosis but neither altered bacterial load nor protected CB17 SCID mice from death. Furthermore, the absence of neutrophils had no impact on the overwhelming systemic inflammatory response in these mice. This response was predominantly driven by activated MΦ and NK cells both of which expressed IFNγ and is considered as the reason of death. Finally, we observed that iNOS expression by MΦ and neutrophils did not correlate with *R*. *typhi* uptake *in vivo*. Moreover, we demonstrate that MΦ hardly respond to *R*. *typhi in vitro*. These findings indicate that *R*. *typhi* enters MΦ and also neutrophils unrecognized and that activation of these cells is mediated by other mechanisms in the context of tissue damage *in vivo*.

## Author Summary

*Rickettsia typhi* causes a relatively mild disease in humans and in immunocompetent mice the bacterium does not cause clinical symptoms as it is easily controlled by the adaptive T cell response. To analyze the role of innate immune mechanisms we here infected mice deficient in T and B cells and find that these mice die within 21 days from a systemic inflammatory response. In addition to splenomegaly due to the accumulation of macrophages and neutrophils, they also show severe liver necrosis that is caused by a massive influx of neutrophils but not the cause of death. The systemic inflammatory response is remarkable, because *R*. *typhi* does not directly activate macrophages and neutrophils. Our study demonstrates a strong immunopathological role of cells of the innate immune system in this infection that may also operate in patients as liver damage is a common symptom of the human disease.

## Introduction

Rickettsioses are emerging febrile diseases that can be fatal. Causative agents are intracellular bacteria of the family of *Rickettsiaceae* that are transmitted to humans by arthropods. The family *Rickettsiaceae* is subdivided into the genera *Rickettsia* and *Orientia*. While the genus *Orientia* has only one member, *Orientia tsutsugamushi* which is the causative agent of scrub typhus, the genus *Rickettsia* is further subdivided into four major groups: The spotted fever group (SFG), the typhus group (TG), the transitional and the ancestral group. The majority of rickettsiae belong to the SFG. Prominent members of this group are *Rickettsia* (*R*.) *rickettsii*, the causative agent of Rocky Mountain Spotted Fever (RMSF), and *R*. *conorii* that causes Mediterranean Spotted Fever (MSF). *R*. *prowazekii* and *R*. *typhi* constitute the typhus group (TG) of rickettsiae [1, 2]. The transitional group consists of *R*. *felis*, *R*. *akari* and *R*. *australis* and members of the non-pathogenic ancestral group are *R*. *bellii* and *R*. *canadensis* [2, 3].

*R*. *prowazekii* and *R*. *typhi* are the causative agents of epidemic and endemic typhus, respectively. These diseases appear with similar symptoms. After an incubation period of 10–14 days the disease starts with the sudden onset of high fever that lasts for several days. Patients further suffer from diverse symptoms including headache, muscle and joint pain, nausea and vomiting. In addition, neurological symptoms such as confusion and stupor are common [4]. As endothelial cells belong to the main target cells of rickettsiae [5], rickettsial infections result in local blood vessel lesions and inflammatory responses. For that reason the majority of patients develop a characteristic hemorrhagic rash as rickettsiae first enter the skin [2]. Systemic infection can result in fatal multi-organ pathology and complications such as pneumonia, myocarditis, nephritis, encephalitis or meningitis [4, 6]. In addition, splenomegaly and liver dysfunction are common [7]. The course of disease of endemic typhus is generally milder than that of epidemic typhus. The lethality of *R*. *typhi* infection is estimated to be <5% [8, 9] while the lethality of *R*. *prowazekii* infection is up to 20–30% [6, 9, 10] if untreated with effective antibiotics such as tetracyclins or chloramphenicol.

Mouse models for rickettsial infections are rare. Immunologically useful strains such as C57BL/6 and BALB/c mice were found to be resistant to various rickettsiae while C3H/HeN mice have been shown to be susceptible [11–15]. Infection of C3H/HeN mice revealed some insight into immune response against rickettsiae in recent years. It has been shown that cytotoxic CD8^+^ T cells in addition to IFNγ are critical for protection against SFG rickettsiae such as *R*. *rickettsii* and *R*. *conorii* in C3H/HeN mice [16–19] while generally little is known about immune response against TG rickettsiae. Mice of the C57BL/6 strain that lack adaptive immunity (C57BL/6 RAG1^-/-^ mice) mount a robust innate immune response that is sufficient to prevent rickettsial disease, at least for a long period of time. C57BL/6 RAG1^-/-^ mice survive the infection with *R*. *conorii* as well as with *R*. *typhi* for at least 20 days [20, 21]. *R*. *typhi*, however, persists in these animals, causing lethal central nervous system inflammation months after infection [21].

In the present study we analyzed CB17 SCID mice in *R*. *typhi* infection. These mice resemble C57BL/6 RAG1^-/-^ mice as they also lack T and B cells [22, 23]. However, whereas C57BL/6 RAG1^-/-^ mice are capable to control the infection for more than 80 days before *R*. *typhi* reappears in the central nervous system, infection of CB17 SCID mice with *R*. *typhi* leads to a complete different outcome. CB17 SCID mice succumbed to *R*. *typhi* infection within 20 days. At the time of death *R*. *typhi* was detectable at high amounts in various organs with the highest bacterial load in the spleen followed by the brain, lung and liver. The most striking pathological changes in CB17 SCID mice were a dramatic enlargement of the spleen and severe liver necrosis. Splenomegaly was mainly due to massive accumulation of MΦ and neutrophils. Both cell types were found to harbor *R*. *typhi* and exhibited an inflammatory and bactericidal phenotype, indicated by the production of reactive oxygen species (ROS) and the expression of inducible nitric oxide synthase (iNOS). We further show that neutrophil-depletion completely prevents liver necrosis in *R*. *typhi*-infected CB17 SCID mice, demonstrating that neutrophil activity is responsible for liver damage in these animals. The absence of neutrophils, however, did not alter bacterial load nor prevent the mice from death. In addition, neutrophil depletion did not influence the strong systemic inflammatory response observed in these mice. This response was dominated by IFNγ, TNFα and IL-6 and mainly driven by MΦ and NK cells. We finally show that MΦ and also neutrophils, although the cells take up *R*. *typhi*, hardly respond to the bacteria in a direct way. iNOS expression by these cells *in vivo* did not correlate with *R*. *typhi* uptake. Moreover, bone marrow-derived MΦ (bmMΦ) were uncapable to kill ingested bacteria and neither released inflammatory mediators nor bactericidal nitric oxide (NO) upon infection with *R*. *typhi in vitro*. These data show that *R*. *typhi* does not activate MΦ in a classical manner although the cells upregulated the expression of major histocompatibility complex class I (MHCI) and CD80. We therefore suggest that MΦ activation in *R*. *typhi*-infected CB17 SCID mice is largely mediated by indirect mechanisms in the context of cellular damage.

Collectively, our data show that liver damage in CB17 SCID mice is due to the action of neutrophils and suggest that overwhelming systemic inflammation is responsible for death of these mice.

## Materials and Methods

### Mice

BALB/c, BALB/c RAG2^-/-^ and congenic CB17 SCID (CB17/lcr-Prkdc^SCID^/lcrlcoCrl) mice that lack T and B cells due to a genetic autosomal recessive mutation in the Prkdc^SCID^ allele on chromosome 16 [22, 23] were bred and maintained in the animal facilities of the Bernhard Nocht Institute for Tropical Medicine, Hamburg, and housed in a biosafety level 3 facility for experimentation. The facilities are registered by the Public Health Authorities (Behoerde für Gesundheit und Verbraucherschutz, Hamburg). All experimentations and procedures were approved by the Public Health Authorities (Behoerde für Gesundheit und Verbraucherschutz, Hamburg: no 88/13) and performed according to the German Animal Welfare Act.

### Culture, purification and freezing of *R*. *typhi*

*R*. *typhi* (Wilmington strain) was cultivated in L929 mouse fibroblasts (ATCC CCL-1) in RPMI1640 (PAA, Cölbe, Germany) supplemented with 10% FCS (PAA, Cölbe, Germany), 2 mM L-glutamine (PAA, Cölbe, Germany) and 10 mM HEPES (PAA, Cölbe, Germany) without antibiotics under biosafety level 3 conditions. 1×10^7^ L929 cells were seeded in 175 cm^2^ culture flasks (Greiner Bio-One, Frickenhausen, Germany) and γ-irradiated (1966 rad at 560sec). One day later cells were infected with *R*. *typhi* and incubated for 5 to 7 days. Stocks of purified bacteria were prepared from L929 cell lysates. Therefore, cells were resuspended in 1.5 ml PBS and vortexed thoroughly for 1 min with 200 μl sterile siliceous particles (60/90 grit silicon carbite, Lortone inc., Mukilteo USA) in a 2 ml SafeSeal tube (Sarstedt, Nümbrecht, Germany). The crude lysate was strained through a 2 μm cell strainer (Puradisc 25 syringe filter 2 μm; GE Healthcare Life Sciences, Freiburg, Germany), mixed in a ratio of 1:1 with 2-fold concentrated storage medium (FCS/15% DMSO) and transferred into Cryo.S tubes (Greiner Bio-One, Frickenhausen, Germany) in liquid nitrogen. Thawed bacterial stocks were centrifuged at 7826xg for 5 min at room temperature, washed once with PBS and analyzed for bacterial content by quantitative real-time PCR (qPCR). Spot forming units (sfu) as a measure for the amount of living bacteria in the preparation were determined by immunofocus assay. For this purpose, L929 cells were incubated with titrated amounts of *R*. *typhi* in 24well plates. After 4h of bacterial adherence the medium was exchange against semi-solid medium containing 1% methylcellulose and cells were further incubated for 8–10 days. For detection of *R*. *typhi*, cells were fixed in PBS/4% formaldehyde/0.1% TritonX100 (Sigma-Aldrich, Deisenhofen, Germany) for 20 minutes followed by permeabilization in PBS/0.5% TritonX100 for 20–60 minutes. Cells were blocked with 200 μl PBS/10% FCS for 1 hour. Monoclonal anti-*R*. *typhi* antibody (BNI52) was added at 1 μg/ml in PBS/10% FCS overnight at 4°C. Cells were washed in H_2_O and with goat anti-mouse HRP (Dako, Hamburg, Germany; 1:400 in PBS/10% FCS) for 1-2h in the dark at RT. Finally, cells were washed and plates were developed with Immunoblot 3,3′,5,5′-tetramethylbenzidine (TMB) substrate solution (Mikrogen, Neuried, Germany; 200 μl) and analyzed with a BZ9000 Keyence microscope (Keyence, Neu-Isenburg, Germany).

### Infection of mice, neutrophil depletion and brefeldin A treatment

Mice were subcutaneously (s.c.) infected into the base of the tail with 2×10^6^ sfu *R*. *typhi* in 50 μl PBS. For neutrophil depletion, 200 μg anti-Ly6G were injected every 3 days intraperitoneally into CB17 SCID mice starting 6 days after infection with *R*. *typhi*. A control group of *R*. *typhi*-infected CB17 SCID received equal amounts of IgG2a isotype antibody. A second control group of animals was not infected and received anti-Ly-6G only. A third group of control mice was not infected and treated with PBS. Depletion of neutrophils was monitored by flow cytometry of blood cells. For flow cytometric detection of intracellular IFNγ and TNFα expression by different cell populations directly *ex vivo* 100 μg brefeldin A (#B7651; Sigma, Deisenhofen, Germany) were intravenously injected into *R*. *typhi*-infected CB17 SCID mice 12h prior to spleen cell isolation. Non-infected control animals that had received PBS instead of *R*. *typhi* were treated the same way. These analyses were performed on day 12 post infection or PBS treatment, respectively.

### Clinical scoring and survival

Based on the findings described in the first part of the results section a clinical score was defined to monitor the health status of *R*. *typhi*-infected animals. The following five criteria were assessed: posture (0: normal, 1: temporarily curved, 2: curved), fur condition (0: normal, 1: staring in the neck, 2: overall staring), activity (0: normal, 1: reduced, 2: strongly reduced), weight loss (0: < 10%, 1: 10–14%, 2: > 15%) and food and water uptake (0: normal, 1: reduced, 2: none). Mice were considered healthy with a score < 5, moderately ill with a score of 5–7 and severely ill with a score of 8–10. Mice were euthanized reaching a total score of ≥8 or showing weight loss of ≥20%. This was determined as the time of death. The state of health of the animals was assessed by clinical scoring every 2 days.

### Collection of blood samples

Blood was taken submandibular or by cardiac puncture after euthanasia with CO_2_. For plasma samples blood was collected in EDTA coated tubes (KABE Labortechnik GmbH, Nümbrecht-Elsenroth, Germany) and centrifuged at 5654×g. Serum samples were obtained by agglutination for 15–20 at RT followed by centrifugation for 10 min 5654×g.

### DNA preparation from purified bacteria, cell culture and organs

10 mg tissue was homogenized in 500 μl PBS in Precellys ceramic Kit tubes (Peqlab. Erlangen, Germany) in a Precellys 24 homogenizer (Peqlab. Erlangen, Germany) with following cycle parameters: 6000 rpm two times for 45 sec with a 60 sec break. DNA was prepared from 80 μl homogenized organs or up to 1×10^6^ L929 cells using the QIAamp DNA Mini Kit (Qiagen, Hilden, Germany) according to the manufacturer’s guide.

### Quantitative real-time PCR (qPCR)

*R*. *typhi* was quantified by amplification of a 137 bp fragment of the *PrsA* gene (RT0565) with the forward primer 5´-ACA GCT TCA AAT GGT GGG GT-3´ and reverse primer 5´-TGC CAG CCG AAA TCT GTT TTG-3´ in a standard SYBR green real-time PCR. To determine *R*. *typhi* copy numbers a standard template plasmid (pCR2.1-PrsA) containing the same *PrsA* DNA fragment was used. Reactions were performed in a Rotor Gene 6000 (Qiagen, Hilden, Germany) in a total volume of 10 μl with 1×HotStar Taq DNA Polymerase Buffer comprising 1.5 mM MgCl_2_, 0.175 mM dNTPs, 100 nM primers, 0.05x SYBR green I nucleic acid gel stain (SIGMA Life Science, Deisenhofen, Germany) and 0.25 U HotStar Taq DNA Polymerase (Qiagen, Hilden, Germany) under following conditions: 15 min pre-heating at 95°C followed by 40 cycles of denaturation (94°C for 20 sec), primer annealing (53°C, 30 sec) and elongation (72°C for 20 sec).

### Antibodies and reagents

Anti-*R*. *typhi* (BNI52) is a monoclonal mouse antibody that was generated at the Bernhard Nocht Institute for Tropical Medicine, Hamburg, Germany. It was used at 1 μg/ml for immunofluorescent stainings of infected cell cultures and flow cytometry. For flow cytometry the following antibodies were used at the indicated dilutions and concentrations: anti-NOS2 (iNOS)-PE (clone CXNFT, 1:200), anti-NOS2 (iNOS)-PE/Cy7 (clone CXNFT, 1:300), anti-MHCI (H-2d)-PE (clone SF1-1.1.1, 1:200), rat IgG2a κ Isotype PerCp-Cy5.5 (eBR2a) and anti-mouse NKp46-PE (clone 29A1.4, 1:200) from eBioscience, Frankfurt, Germany; anti-CD11b-PerCp-Cy5.5 (clone M1/70, 1:200; 1:800 for bmMΦ) from BD Bioscience, Heidelberg, Germany; anti-Ly6-C-PerCP/Cy5.5 (clone HK1.4, 1:200), anti-Ly-6G-APC (clone 1A8, 1:166,7), anti-GR1 (L6G/Ly6C)-APC (clone GR-1/RB6-8C5, 1:500), anti-CD80-PE/Dazzle594 (clone 16-10A1, 1:100), anti-CD11b-FITC (clone M1/70, 1:100), rat IgG2a κ isotype PE (RTK2758; 1:200), rat IgG1 κ isotype PE-Cy7 (RTK20711; 1:200), anti-mouse IFNγ PE/Dazzle (clone XMG1.2; 1:333), anti-mouse TNFα BV510 (clone MP-6-XT22; 1:80) and rat IgG1 κ isotype PE/Dazzle 594 (RTK 2071; 1:200) from BioLegend (London, UK); unlabeled mouse IgG3 κ isotype (clone B10; 1 μg/ml) and anti-mouse-IgG3-FITC from SouthernBiotech (#1100–02; Birmingham, USA; 1:200). Histological stainings were performed with the following antibodies and reagents: *R*. *typhi* patient serum (1:100) from the diagnostics department of the Bernhard Nocht Institute for Tropical Medicine, Hamburg, Germany; anti-mouse iNOS (ABIN373696, 1:75) from Abcam, Cambridge, USA; anti-mouse IBA1 (#019–19741; 1:500) from WAKO, Neuss, Germany; anti-mouse Ly-6G (clone 1A8; 1:1000) from BD Biosciences, Heidelberg, Germany; anti-mouse-IgG3-FITC (#1100–02; 1:200) from SouthernBiotech, Birmingham, USA; anti-human IgG-FITC (#H10101C; 1:200), anti-rabbit Alexa555 (#A31572; 1:300) and anti-rat Alexa568 (#A11077; 1:300) from Thermo Fisher Scientific, Braunschweig, Germany; anti-FITC-Alexa488 (1:1000) from LifeTechnologies, Darmstadt, Germany; DAPI (4´,6-diamidino-2-phenylindole dihydrochloride; 1:1000) and CohnII human IgG fraction (5% in PBS) from Sigma, Deisenhofen, Germany. Isotype antibodies were used at concentrations corresponding to the respective staining antibodies. For neutrophil depletion anti-Ly-6G (clone 1A8) and rat IgG2a (clone 2A3) isotype control antibody (BioXCell, New Hampshire, USA) were used.

### Flow cytometry

Single cell suspensions were prepared from spleen, liver, blood samples or cell culture. Erythrocytes were eliminated from spleen, liver and blood samples by incubating the cells in erythrocyte lysis buffer (10 mM Tris, 144 mM NH_4_Cl, pH 7.5) for 5 minutes at room temperature. Afterwards, cells were washed twice with PBS. Fc receptors were blocked with 50 μl 5% CohnII human IgG fraction (Sigma-Aldrich, Deisenhofen, Germany) in PBS or Perm/Wash solution. BD Cytofix/Cytoperm and BD Perm/Wash solutions (BD Biosciences, Heidelberg, Germany) were used for intracellular staining of iNOS and *R*. *typhi*. Intracellular cytokines were detected employing the FoxP3/Transcription Factor Staining Buffer Set (eBioscience, Frankfurt, Germany). Procedures were performed according to the manufacturer´s instructions. CD80 and MHCI on bmMΦ were first stained extracellularly followed by intracellular staining of iNOS and *R*. *typhi*. Antibodies were diluted in 50 μl of either PBS or Perm/Wash solution. Cytokines (TNFα and IFNγ), NKp46, CD11b and GR1 were stained simultaneously intracellularly in Permeabilization Buffer of the FoxP3/Transcription Factor Staining Buffer Set. After staining, cells were washed and resuspended in PBS/1% PFA or PBS/10% FCS prior to flow cytometry. Analyses were performed with a BD Accuri C6 or BD LSR II flow cytometer (BD Biosciences, San José, USA) and FlowJo single cell analysis software (FlowJo LLC, Ashland, USA).

### Detection of reactive oxygen species (ROS)

ROS release was determined in blood cells of infected mice by the formation of the fluorescent compound rhodamine-123 from dihydrorhodamine-123 (DHR-123, AAT Bioquest, California, USA). Staining was performed with 30 μl EDTA blood samples after erythrocyte lysis. Surface markers were stained as described above prior to ROS detection. Cells were incubated with 30 μg/ml DHR-123 in PBS for 20 min at 37°C in the dark. After washing with 4 ml of cold PBS 40 μl of cell suspensions were immediately analyzed using BD Accuri C6 (BD Biosciences, San José, USA) and FlowJo single cell analysis software (FlowJo LLC, Ashland, USA).

### Detection of cytokines in plasma and cell culture supernatants

Plasma cytokines were quantified by bead-based LEGENDplex immunoassay (BioLegend, London, UK) according to the manufacturer’s protocol using cluster tubes (ThermoScientific, Loughborough UK). 12.5 μl of plasma from EDTA blood samples was used diluted 1:2 in PBS. Supernatants from bmMΦ were used non-diluted. Analyses were performed using a BD Accuri C6 (BD Biosciences, San José, USA) and LEGENDplex analysis software (BioLegend, San Diego, USA).

### Detection of serum Glutamate pyruvate transaminase (GPT)

Serum levels of GPT were evaluated using Reflotron GPT (ALT) stripes and Reflotron Plus device (Roche Diagnostics, Mannheim, Germany) according to the manufacturer’s instructions. Serum samples were diluted 1:3 in PBS prior to analyses.

### Generation and infection of bmMΦ *in vitro*

Bone marrow was isolated from femur and tibia of BALB/c mice. 2×10^6^ cells were differentiated in petri dishes for 12 days in IMDM (PAA, Cölbe, Germany) supplemented with 10% FCS, 2 mM L-glutamine, 5% horse serum (Biochrom, Berlin, Germany) and L929 fibroblast medium as a source of M-CSF. Fresh medium was applied every 3 days. After 12 days of differentiation bmMΦ were harvested and washed twice with PBS. For analysis of NO and cytokine release as well as flow cytometry, bmMΦ were seeded into 24-well tissue culture plates at 5×10^5^ cells per well and infected in duplicates with 5, 10 or 25 *R*. *typhi* particles as determined by qPCR per cell. Control cells were incubated with medium or stimulated with 0.5 μg/ml *E*. *coli* (strain 055:B5) lipopolysaccharide (LPS) (Sigma, Deisenhofen, Germany). After incubation of 24 and 48 hours cells were analyzed by qPCR and flow cytometric staining of *R*. *typhi*, MHCI, CD80 and iNOS. Cytokines and nitric oxide (NO) were quantified in the supernatants as described below.

### Detection of NO

NO concentrations were determined by Griess reaction in supernatants of bmMΦ. Assays were performed in microtiter plates (Greiner Bio-One, Frickenhausen, Germany). 100 μl of sample were mixed with 50 μl Griess 1 reagent (0.5 g sulfonamide in 50 ml 1M HCl) and 50 μl Griess 2 reagent (0.15 g naphtylethylendiamine-dihydrochloride in 50 ml H_2_O). A serial dilution of sodium nitrite (NaNO_2_) in culture medium was used as a standard (c_max_ 125 μM). The absorbance was measured at 560 nm with a Dynex MRXII spectrophotometric microplate reader (Dynex Technologies, Chantilly, USA).

### Immunofluorescence staining of *R*. *typhi* in bmMΦ

5×10^4^ bmMΦ were seeded into 8well Nunc Permanox chamber slides (Sigma-Aldrich, Munich, Germany). Living *R*. *typhi* or heat-inactivated *R*. *typhi* particles (30 min, 56°C) were added at 10 copies per cell. Control cells were not infected. Medium was exchanged after 4 h of bacterial adherence. Cells were further incubated for 48 h and permeabilized by addition of ice-cold acetone:methanol (1:3) for 10 minutes at -20°C. Cells were washed 3 times in PBS and staining procedures were then performed at 37°C. Fc receptors were blocked with 5% CohnII in PBS for 15 minutes and 1 μg/ml anti-*R*. *typhi* (BNI52) was added for additional 30 minutes. After washing, cells were stained with anti-mouse IgG3-FITC (1:200) in PBS for 30 minutes followed by staining with anti-FITC-Alexa488 and DAPI (both 1:1000). Finally, cells were washed in PBS and slides were covered with Permafluor Mounting Medium and cover slips (ThermoScientific, Loughborough UK). Images were taken with the Axioskop MC-80 microscope (Zeiss, Oberkochen, Germany).

### Histological stainings

For immunohistochemistry (IHC) tissues from infected mice were fixed in 4% formalin in PBS and embedded in paraffin. Deparaffinization of the sections was performed using standard methods. Sections were first heated at 63°C for 30 minutes in a heating cabinet followed by treatment with Xylol for 30 minutes and EtOH (3x 100% EtOH, 3x 96% EtOH, 80% EtOH, 70% EtOH). Each step was performed for 3–5 minutes. Slides were finally washed in H_2_O. Deparaffinized sections were boiled for 30 minutes in 10 mM citrate buffer (10 mM sodium citrate, 0.05% Tween20, pH6.0) for antigen retrieval. Staining was performed using a Ventana Benchmark XT apparatus (Ventana, Tuscon, USA). Antibodies were diluted in 5% goat serum (Dianova, Hamburg, Germany) in Tris-buffered saline pH7.6 (TBS) and 0.1% Triton X100 in antibody diluent solution (Zytomed, Berlin, Germany). Rabbit anti-mouse IBA1 (1:500), rabbit anti-mouse iNOS (1:75) and rat anti-mouse Ly-6G (1:1000) were used. *R*. *typhi* was detected employing serum from a *R*. *typhi*-patient (1:100). Slides were incubated with primary antibodies for 1 h. Histofine Simple Stain MAX anti-human, anti-rabbit, anti-mouse or anti-rat peroxidase-coupled antibodies (Nichirei Biosciences, Tokyo, Japan) were used as secondary antibodies. Detection was performed with ultraview universal DAB detection kit (Ventana, Tuscon, USA). For immunofluorescent stainings donkey anti-rabbit Alexa555 (1:300), goat anti-rat Alexa568 (1:300), goat anti-human IgG-FITC (1:200) and anti-Alexa488-FITC (1:1000) were used as secondary antibodies. Nuclei were stained with DAPI (1:1000). Sections were covered with Tissue-Tek embedding medium (Sakura Finetek, Staufen, Germany). Images were taken with a BZ9000 Keyence microscope (Keyence, Neu-Isenburg, Germany).

### Statistical analyses

Statistical analyses were performed with GraphPad Prism 5 software (GraphPad Software, Inc., La Jolla, USA). The proportion of surviving animals was analyzed with the Log-rank Mantel Cox test. Normality test was performed with D'Agostino-Pearson normality test for n≥8. For comparison between two groups two-tailed Students t-test for parametric samples or Mann-Whitney U test for non-parametric samples were applied. To assess differences between multiple groups Kruskal-Wallis test followed by Dunn´s post-test or Two way ANOVA followed by Tukey´s post- est were applied.

## Results

### T and B cell-deficient CB17 SCID mice are highly susceptible to *R*. *typhi* and show disseminated infection

**Fig 1:**
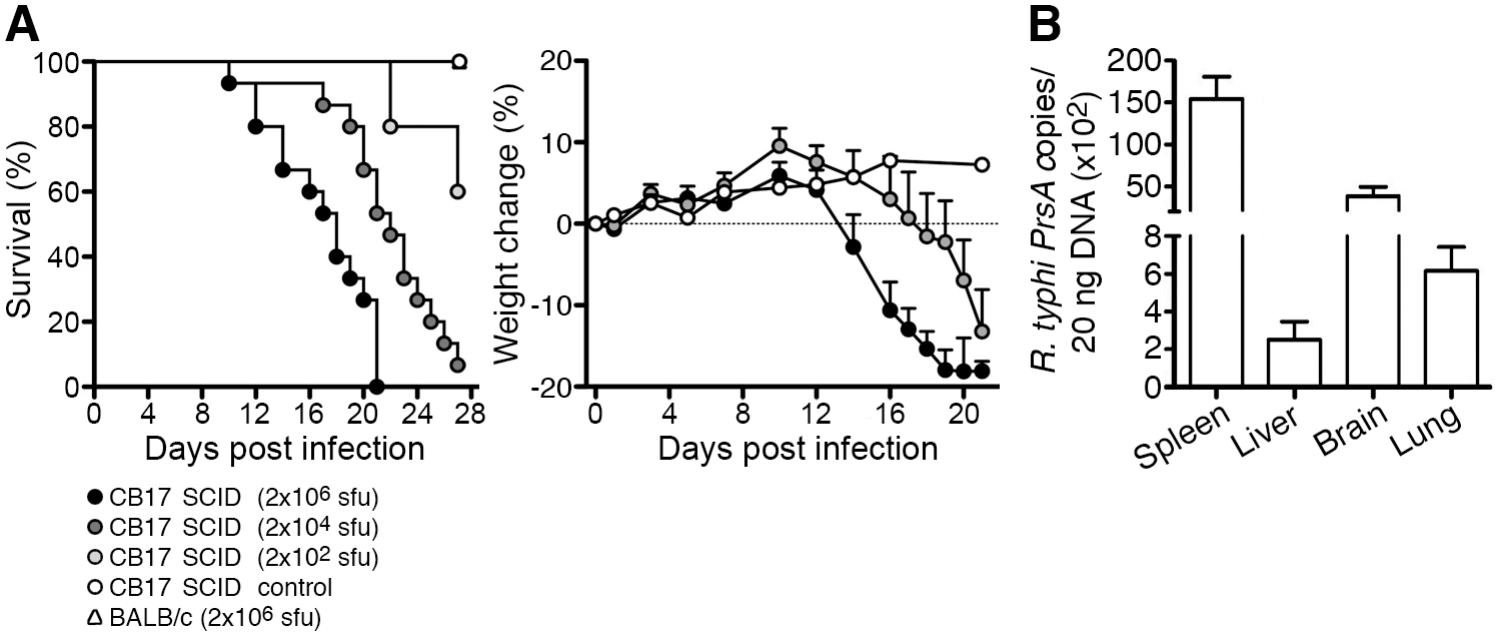
CB17 SCID mice succumb to *R*. *typhi* infection and develop systemic infection. CB17 SCID mice were infected s.c. with 2×10^6^, 2×10^4^ or 2×10^2^ sfu *R*. *typhi* into the base of the tail (n = 10 for each group). BALB/c mice received 2×10^6^ sfu *R*. *typhi* via the same route (n = 11). Control CB17 SCID mice received PBS (n = 10). Survival rates (left; y-axis) and weight loss were monitored (right; y-axis) (**A**). Bacterial burden (y-axis) was determined in liver, spleen, brain and lung of CB17 SCID after s.c. infection with 2×10^6^ sfu *R*. *typhi* (n = 10–11) by *PrsA* qPCR at the time of death (**B**). Data represent combined results from two independent experiments and show the mean ± SEM.

To determine the susceptibility of CB17 SCID mice to *R*. *typhi* infection, we infected the animals with titrated amounts of *R*. *typhi* (2×10^6^, 2×10^4^ and 2×10^2^ sfu) s.c. into the base of the tail. A control group of CB17 SCID mice received PBS instead of *R*. *typhi*. Immunocompetent congenic BALB/c wild-type mice were infected with the highest dose of *R*. *typhi* as an additional control. As expected, BALB/c wild-type mice did not show clinical symptoms of disease at any point in time and all animals survived the infection (Fig 1A and S3B Fig). In contrast, CB17 SCID mice were highly susceptible. 100% of the mice that received 2×10^6^ sfu and 94% of the mice that were infected with 2×10^4^ sfu succumbed to the infection while 60% of the mice that obtained 2×10^2^ sfu survived the infection (Fig 1A). The survival period was 16.93±3.75 days for mice that were infected with the highest dose, 22.07±2.74 days for mice infected with the median dose and 24.5±3.54 days for those that received the lowest dose. Staring fur was the first sign of illness and appeared around day 7–10 post infection in animals that received the highest dose of *R*. *typhi* (2×10^6^ sfu) while the onset of disease was later in mice that were infected with 2×10^4^ (day 12–14). Disease then rapidly progressed and the animals showed hunchback appearance and inactivity. This correlated with body weight loss that further progressed until death while PBS-treated CB17 SCID control mice remained healthy and gained weight (Fig 1A). Based on these findings the lethal dose of 2×10^6^ sfu was used for all further experimentation.

Next, bacterial dissemination was analyzed. Bacterial load was determined in liver, spleen, brain and lung by qPCR. High amounts of *R*. *typhi* were found in all organs in CB17 SCID mice at the time of death. The highest bacterial load was detected in spleen followed by the brain, lung and liver (Fig 1B), demonstrating disseminated infection in these animals. These results show that CB17 SCID mice are highly susceptible to *R*. *typhi* infection and fail to control bacterial growth.

### *R*. *typhi*-infected CB17 SCID mice develop splenomegaly which is predominantly caused by the accumulation of neutrophils and MΦ

**Fig 2:**
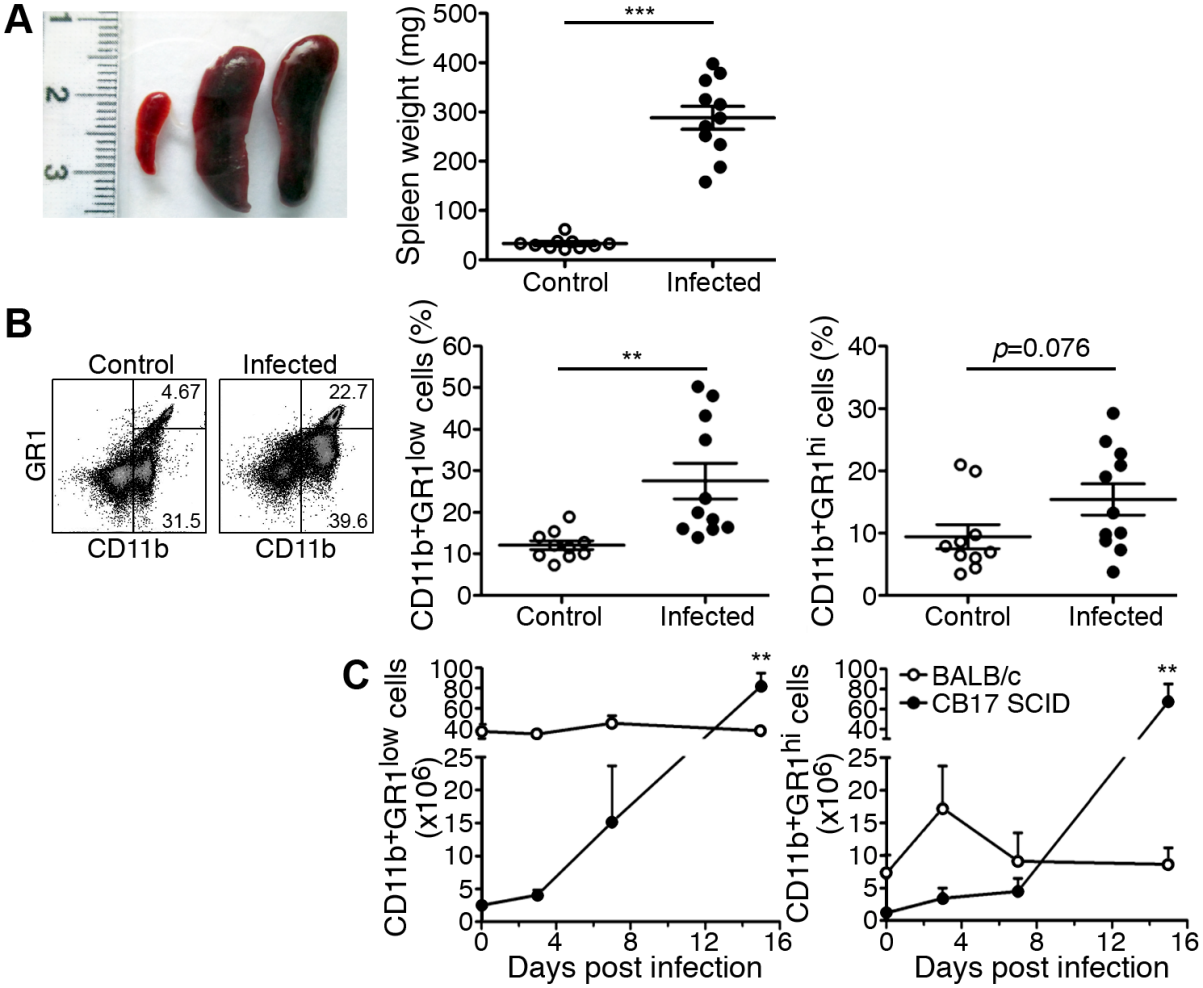
CB17 SCID mice develop splenomegaly which is largely due to the accumulation of MΦ and neutrophils. CB17 SCID mice were infected s.c. with 2×10^6^ sfu into the base of the tail. Control CB17 SCID mice received PBS. The photograph shows a representative spleen of a control mouse (left) and two *R*. *typhi*-infected CB17 SCID mice at the time of death (middle and right). Spleen weight (y-axis) was determined. The graph shows combined results from two independent experiments. Each dot represents a single mouse (n = 10–11). The mean ± SEM is presented. Data were analyzed with unpaired t test. Asterisks indicate statistically significant differences (***p<0.001) (**A**). The percentage of CD11b^+^GR1^low^ MΦ/monocytes and CD11b^+^GR1^hi^ neutrophils among spleen cells (y-axis) in CB17 SCID control animals (open circles) and *R*. *typhi*-infected CB17 SCID mice (black circles) was assessed at the time of death of the animals by flow cytometry. A representative staining for CD11b and GR1 and gating of cells is shown for a control mouse and a *R*. *typhi*-infected CB17 SCID mouse. Numbers indicate the percentage of the CD11b^+^GR1^hi^ neutrophils and CD11b^+^GR1^low^ MΦ/monocytes. Graphs show combined results from two independent experiments. Each dot in the graphs represents a single mouse (n = 10–11). The mean ± SEM is depicted. Statistical analysis was performed with student`s t-test after D´Agostino and Pearson normality test. Asterisks indicate statistically significant differences (** *p*<0.01) (**B**). BALB/c (n = 5–7) and CB17 SCID mice (n = 5–7) were infected s.c. with 2×10^6^ sfu into the base of the tail. Total numbers of CD11b^+^GR1^low^ MΦ/monocytes and CD11b^+^GR1^hi^ neutrophils among spleen cells (y-axis) were assessed by flow cytometric staining as described above during the course of infection. Graphs show the total numbers of CD11b^+^GR1^low^ MΦ/monocytes and CD11b^+^GR1^hi^ neutrophils (y-axis) at the indicated point in time (x-axis). The mean ± SEM is depicted. Statistical analysis was performed with Kruskal-Wallis test followed by Dunn´s post-test comparing samples from infected mice with samples from control animals (day 0) (**C**).

We further investigated pathological changes in CB17 SCID mice. *R*. *typhi*-infected CB17 SCID mice showed a very strong increase in spleen size and weight (288.2±23.3 mg) at the time of death compared to 33.1±2.6 mg in control CB17 SCID mice (Fig 2A). The cellular composition of the spleen of *R*. *typhi*-infected CB17 SCID mice was further assessed by flow cytometric analysis. Cells were stained for CD11b and GR1 to distinguish CD11b^+^GR1^hi^ neutrophils from CD11b^+^GR1^low^ MΦ/monocytes as depicted in Fig 2B (left). *R*. *typhi*-infected CB17 SCID mice showed a significantly increased frequency of MΦ/monocytes. At the time of death CD11b^+^GR1^low^ MΦ/monocytes constituted 27.6±4.3% of the spleen cells compared to 12.1±1.1% in CB17 SCID control mice (Fig 2B, middle). A similar trend was true for CD11b^+^GR1^hi^ neutrophils that represented 9.5±1.9% of the cells in the spleen of control mice and 15±2.5% of the spleen cells in *R*. *typhi*-infected CB17 SCID mice (Fig 2B, right). Thus, together MΦ/monocytes and neutrophils represented approximately 40% of the spleen cells.

We further analyzed the absolute numbers of MΦ/monocytes and neutrophils in the spleen during the course of infection and compared CB17 SCID mice and BALB/c wild-type mice. In BALB/c wild-type mice numbers of CD11b^+^GR1^low^ MΦ/monocytes remained unaltered during the course of infection (Fig 2C, left) while numbers of CD11b^+^GR1^hi^ neutrophils were slightly enhanced early in infection on day 3 (control: 7.22×10^5^, *R*. *typhi*-infected: 1.73×10^6^) and returned to basal counts until day 7 (Fig 2C, right). In *R*. *typhi*-infected CB17 SCID mice numbers of CD11b^+^GR1^hi^ neutrophils and CD11b^+^GR1^low^ MΦ/monocytes steadily increased during the course of infection beginning on day 3 and resulting in an approximately 30-fold increase of MΦ/monocytes (control: 2.5×10^5^, *R*. *typhi*-infected: 8.2×10^6^; Fig 2C, left) and 50-fold increase of neutrophils immediately prior to death (control: 1.18×10^5^, *R*. *typhi*-infected: 6.74×10^6^; Fig 2C, right).

These results demonstrate that splenomegaly in *R*. *typhi*-infected CB17 SCID mice is largely due to disproportionate increase of MΦ/monocytes and neutrophils.

### CB17 SCID mice develop severe liver necrosis upon *R*. *typhi* infection

**Fig 3:**
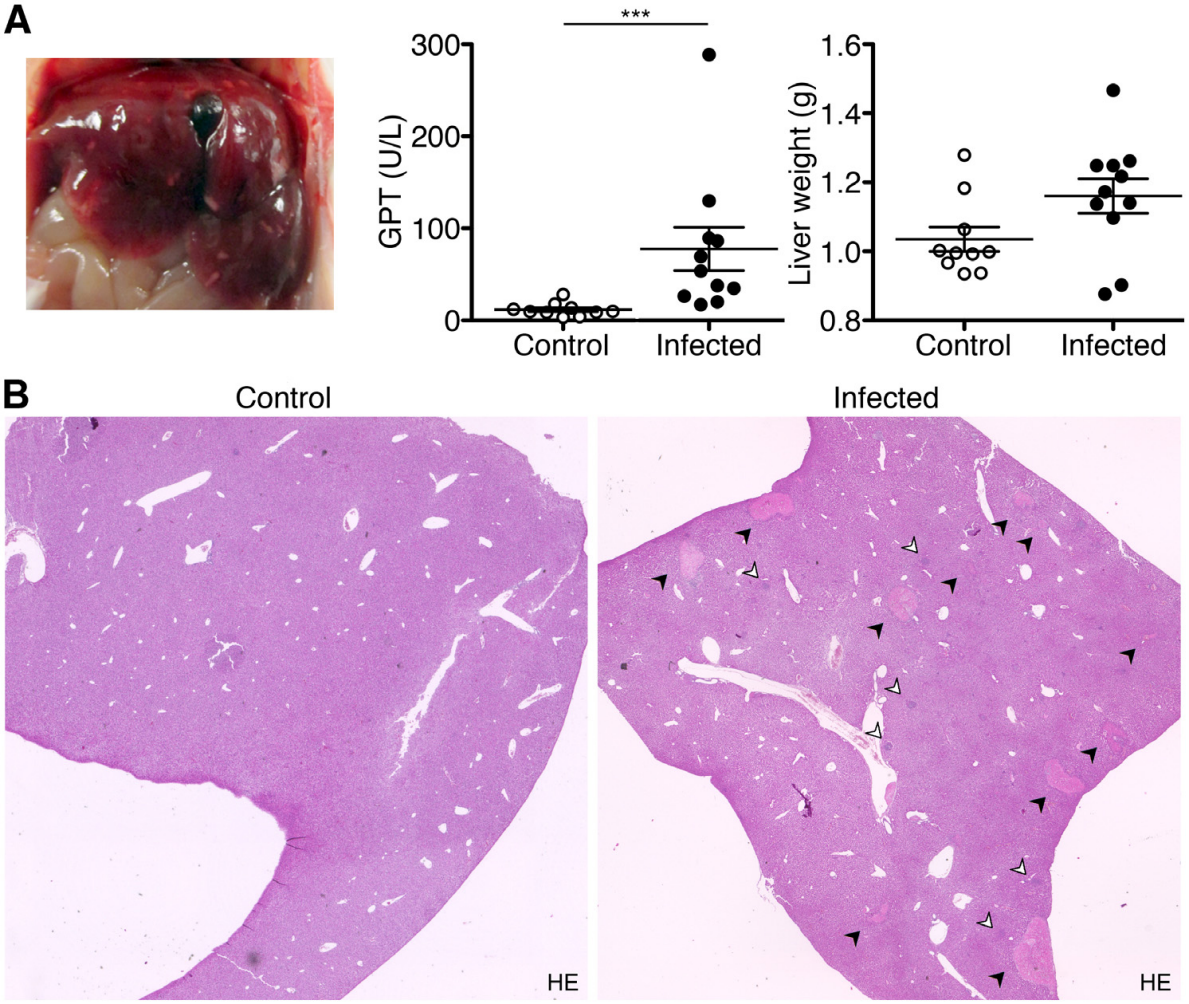
*R*. *typhi*-infected CB17 SCID mice develop severe liver necrosis. CB17 SCID mice were infected s.c. with 2×10^6^ sfu into the tail base while control mice received PBS. The photograph shows a representative liver of a *R*. *typhi*-infected CB17 SCID mouse at the time of death (left). GPT was measured in sera (y-axis) of control mice (open symbols) and *R*. *typhi*-infected CB17 SCID mice (black symbols) (middle) and liver weight (y-axis) was determined at the time of death (right). The graphs show combined results from two independent experiments (n = 10). Each dot represents a single mouse. The mean ± SEM is given. Statistical significance was analyzed by Mann Whitney U test after D´Agostino and Pearson normality test (**A**). Representative histological stainings of the liver from a control mouse (left) and a *R*. *typhi*-infected CB17 SCID mouse (right) with HE are depicted. Images were taken at 2-fold magnification. Black arrows point to necrotic lesions. White arrows indicate foci of infiltrating cells (**B**).

**Fig 4:**
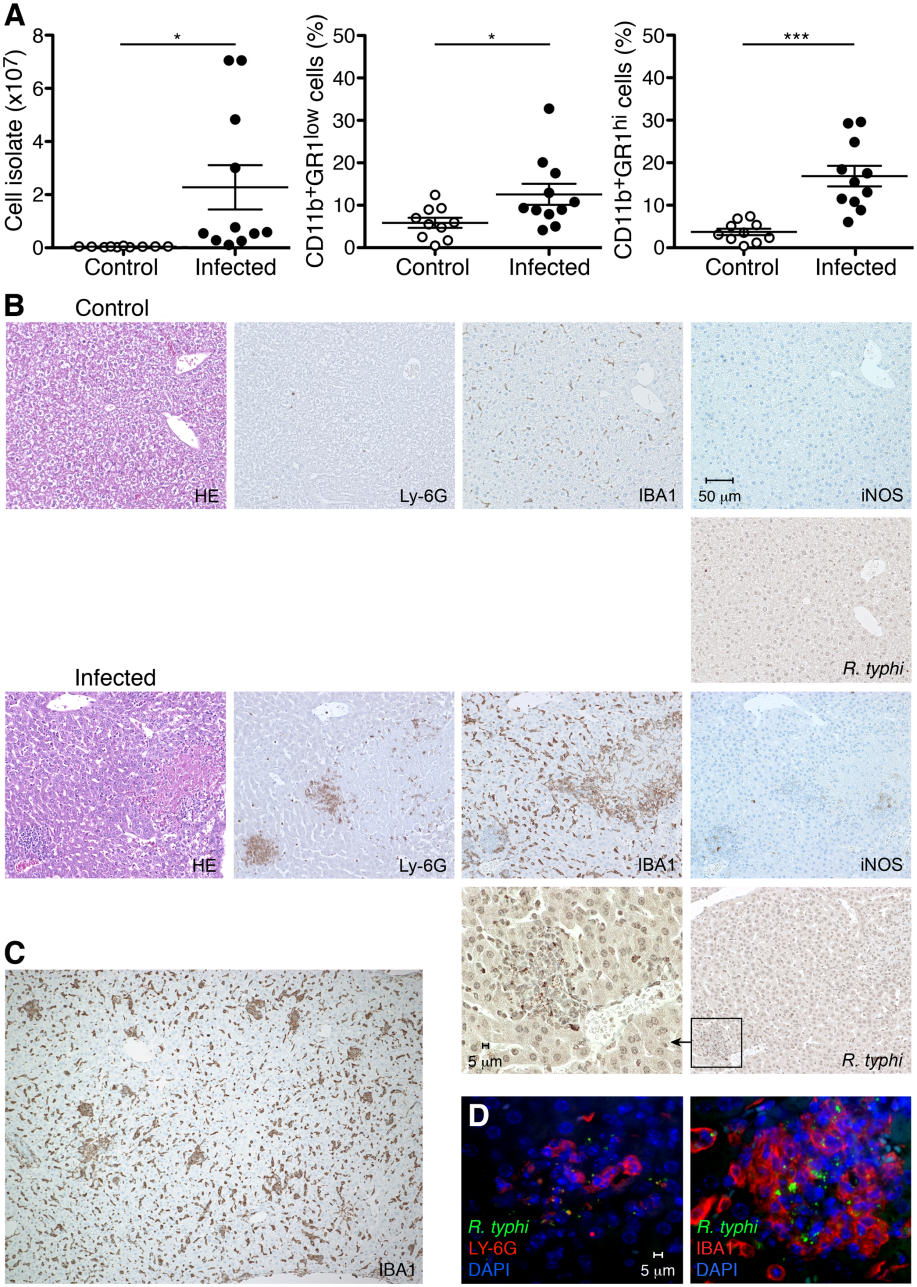
CD11b^+^GR1^low^ MΦ/monocytes as well as of CD11b^+^GR1^hi^ neutrophils infiltrate the liver. CB17 SCID mice were infected s.c. with 2×10^6^ sfu into the base of the tail. Control mice received PBS. At the time of death of *R*. *typhi*-infected mice the numbers of cellular isolates from liver (left) and the percentage of CD11b^+^GR1^low^ MΦ/monocytes (middle) and CD11b^+^GR1^hi^ neutrophils (right) (y-axis) were determined by flow cytometry. Graphs show combined results from two independent experiments and each dot represents a single mouse (n = 10–11). The mean ± SEM is depicted. Data were analyzed by Student`s t-test (left and right) or Mann Whitney test (middle) after D´Agostino and Pearson normality test. Asterisks indicate statistically significant differences (*p<0.05, *** *p*<0.001) (**A**). Serial sections of the liver from a control mouse and a *R*. *typhi*-infected CB17 SCID mouse were stained for IBA1, Ly-6G, iNOS and *R*. *typhi* as indicated. Pictures show a necrotic area. (**B**). In addition, several foci of infiltrating IBA1^+^ MΦ from the periphery were observed (**C**). Immunofluorescent co-stainings were performed for *R*. *typhi* (green) and Ly-6G or IBA1 (red) from the liver of an infected CB17 SCID mouse. Nuclei were stained with DAPI (blue) (**D**).

Next to the massive enlargement of the spleen *R*. *typhi*-infected CB17 SCID mice developed severe liver necrosis which was already visible by eye. In addition, the gall bladder of *R*. *typhi*-infected CB17 SCID mice was dark, indicating endothelial damage and bleedings in the organ (Fig 3A, left). Liver damage was measurable by significantly elevated levels of GPT in the serum of *R*. *typhi*-infected CB17 SCID mice (Fig 3A, middle). In addition, liver weight was enhanced in several animals at the time of death although these differences were not significant (1162±166.8 mg compared to 1035±11.7 mg in control mice; Fig 3A, right). Numerous necrotic areas were visible in histological stainings of the liver of *R*. *typhi*-infected CB17 SCID mice compared to control animals that received PBS instead of bacteria and several spots of infiltrating cells were detectable that were not present in the liver of healthy control mice (Fig 3B). Cellular infiltration began around day 7 post infection when the bacteria were predominantly found in endothelial cells and necrotic lesions were still absent. Infiltrating cells further increased until death around day 15. At this point in time the bacteria were detectable in foci of cellular infiltrates (S1 Fig). Further flow cytometric analysis of cellular isolates from the liver at the time of death confirmed a significant increase of cellular infiltrates (Fig 4A, left). Among these a significantly enhanced percentage of both CD11b^+^GR1^low^ MΦ/monocytes (12.6±2.5% compared to 5.9±1.2% in control mice; Fig 4A, middle) and CD11b^+^GR1^hi^ neutrophils (16.9±2.4% compared to 3.7±0.7% in control mice; Fig 4A, right) was observed, demonstrating that MΦ/monocytes as well as neutrophils infiltrate the liver to a comparable extent.

We further performed serial histological sections to clarify the localization of these cells as well as of *R*. *typhi* in the liver. For this purpose sections were stained for IBA1 which is exclusively expressed by MΦ [24] and Ly-6G as a marker for granulocytes [25]. In addition, iNOS was stained as an indicator for cellular activation. Enhanced numbers of IBA1^+^ MΦ were found equally distributed in the liver parenchyma of *R*. *typhi*-infected CB17 SCID mice, indicating hyperplasia of Kupffer cells. IBA1^+^ MΦ accumulated around necrotic areas (Fig 4B). In addition, several foci of infiltrating IBA1^+^ MΦ from the periphery were observed (Fig 4C). In contrast to MΦ, infiltrating Ly-6G^+^ granulocytes almost exclusively clustered in foci often found nearby necrotic regions (Fig 4B). Expression of iNOS was detectable in IBA1^+^ MΦ as well as in Ly-6G^+^ neutrophils (Fig 4B), demonstrating an activated phenotype of both cell populations. *R*. *typhi* was detectable in clusters of infiltrating Ly-6G^+^ neutrophils as well as IBA1^+^ MΦ but not within necrotic tissue (Fig 4B and 4D).

### Neutrophils and MΦ harbor *R*. *typhi* and show an activated phenotype

**Fig 5:**
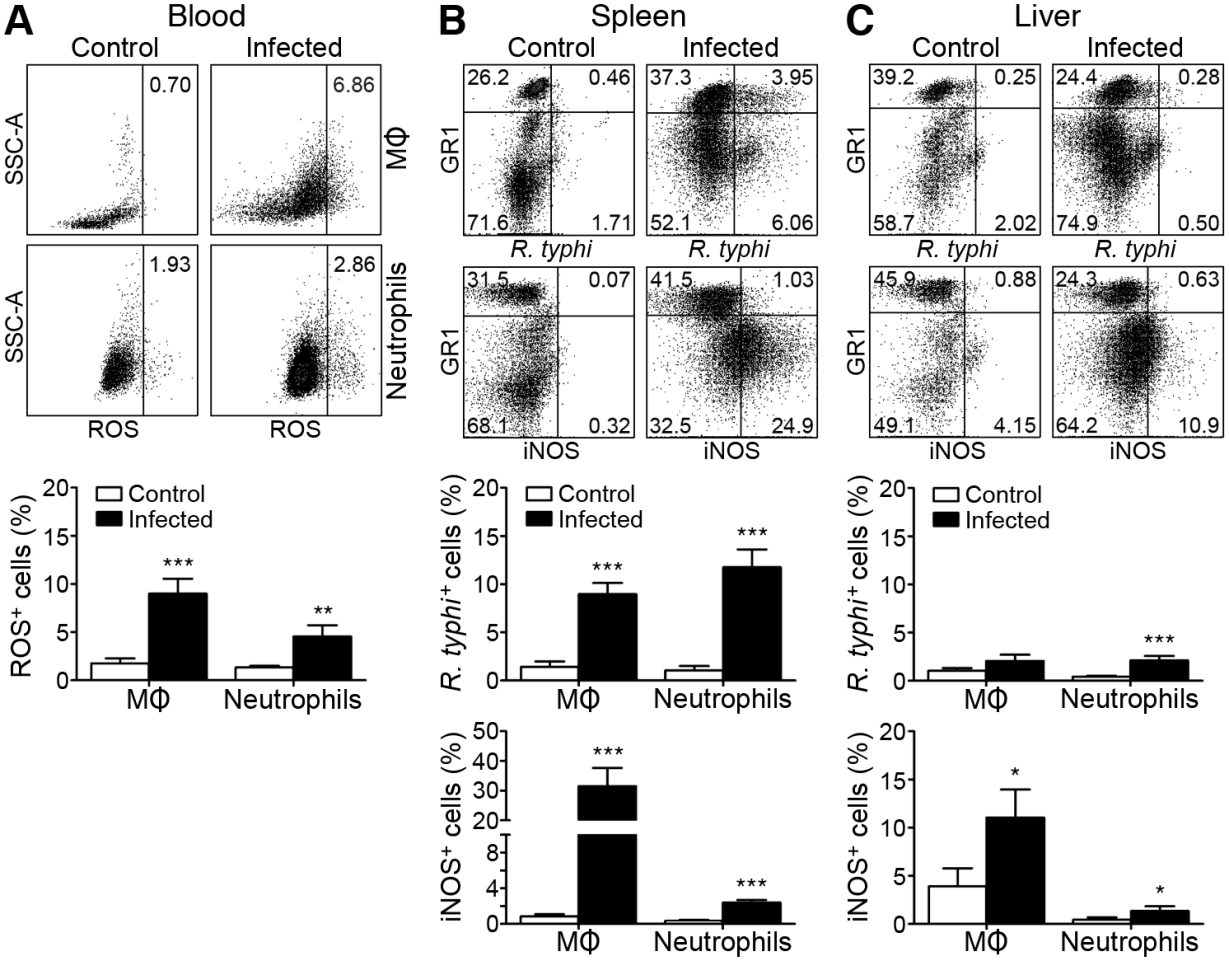
Neutrophils and MΦ release ROS and express iNOS. CB17 SCID mice were infected s.c. with 2×10^6^ sfu into the base of the tail. At the time of death blood, spleen and liver cell samples were analyzed by flow cytometry for ROS release, *R*. *typhi* uptake and iNOS expression. A representative dot plot of ROS staining in CD11b^+^Ly-6C^+/-^Ly-6G^-^ MΦ/monocytes (upper panel) and CD11b^+^Ly-6G^+^ neutrophils (lower panel) from a control mouse (left) and a *R*. *typhi*-infected mouse (right) is shown. The graph depicts the mean ± SEM of the percentage of ROS-releasing cells (y-axis) among MΦ/monocytes and neutrophils (x-axis) determined in blood samples from two independent experiments (n = 10–11) (below) (**A**). Representative dot plots of spleen (**B**) and liver cells (**C**) gated on CD11b^+^ cells and further stained for GR1, intracellular *R*. *typhi* (upper panel) and iNOS (lower panel) are shown. Graphs show the percentage of *R*. *typhi*^+^ and iNOS^+^ cells (y-axis) among CD11b^+^GR1^low^ MΦ/monocytes and CD11b^+^GR1^hi^ neutrophils (x-axis). Data show the mean ± SEM of combined results from two independent experiments (n = 10–11). Statistical analysis was performed with Mann-Whitney U test (*p<0.05, **p<0.01, ***p<0.001) (**B**,**C**).

Activated phagocytes that exert bactericidal functions release reactive oxygen species (ROS) and express iNOS to produce nitric oxide (NO) [26]. To further assess these functional properties of MΦ/monocytes and neutrophils in *R*. *typhi* infection we performed flow cytometric analyses. CD11b^+^GR1^low^ MΦ/monocytes and CD11b^+^GR1^hi^ neutrophils from *R*. *typhi*-infected CB17 SCID mice were analyzed at the time of death of the animals for ROS release, iNOS expression and bacterial uptake. Non-infected mice that received PBS instead of *R*. *typhi* were used as a control. Fig 5A (left) shows a representative flow cytometric analysis of ROS content in CD11b^+^GR1^low^ MΦ/monocytes (top) and neutrophils (bottom) in the blood. ROS were detectable in around 7% of the CD11b^+^GR1^low^ MΦ/monocyte population and 3% of the CD11b^+^GR1^hi^ neutrophils in *R*. *typhi*-infected CB17 SCID mice (Fig 5A). We further analyzed bacterial uptake and iNOS expression in CD11b^+^GR1^low^ MΦ/monocytes and CD11b^+^GR1^hi^ neutrophils in spleen and liver. Representative dot plots of intracellular staining of *R*. *typhi* and iNOS in cells from both organs are shown in Fig 5B and 5C (top). *R*. *typhi* was detectable in a high proportion of CD11b^+^GR1^low^ MΦ/monocytes (9.0±1.2% in the spleen and 2.1±0.7% in the liver) and CD11b^+^GR1^hi^ neutrophils (11.8±1.9% in the spleen and 2.1±0.5% in the liver), demonstrating ingestion of *R*. *typhi* by both cell populations (Fig 5B and 5C). Furthermore, CD11b^+^GR1^low^ MΦ/monocytes as well as CD11b^+^GR1^hi^ neutrophils expressed iNOS in spleen (31.5±6.2% of CD11b^+^GR1^low^ MΦ/monocytes and 2.4±0.3% of CD11b^+^GR1^hi^ neutrophils) and liver (11.1±2.9% of CD11b^+^GR1^low^ MΦ/monocytes and 1.3±0.5% of CD11b^+^GR1^hi^ neutrophils) with MΦ representing the main iNOS-expressing cell population in both organs (Fig 5B and 5C). Thus, MΦ and neutrophils exhibit a bactericidal phenotype.

### Neutrophil depletion neither alters the course of infection nor bacterial load

**Fig 6:**
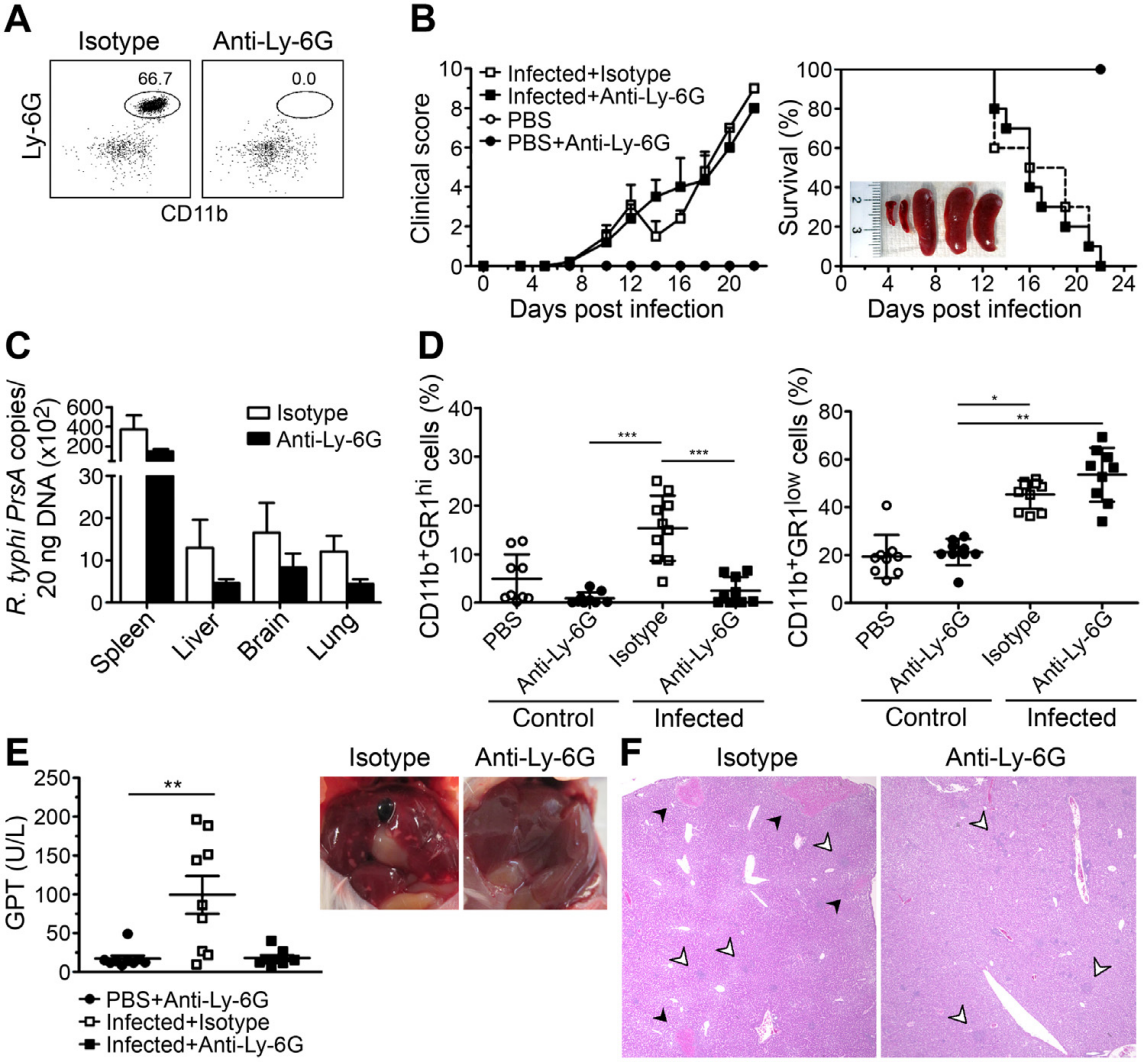
Neutrophil depletion completely prevents liver damage in CB17 SCID mice but does not protect from death. CB17 SICD mice were infected s.c. with 2×10^6^ sfu into the base of the tail. Neutrophils were depleted by intraperitoneal application of anti-Ly-6G antibody. Control animals received rat IgG2a isotype antibody. Depletion was first performed on day 6 post infection and then repeated every 3 days. Efficacy of depletion was assessed by flow cytometric staining of blood cells 1 day after the second depletion (day 10 post infection). A representative dot plot of blood cells from an isotype antibody-treated mouse (left) and a neutrophil-depleted mouse (right) stained for Ly-6G and CD11b is depicted (**A**). The health status of the mice was assessed over time (x-axis) using a clinical score (y-axis). Four groups of mice were analyzed (non-infected mice that were treated with PBS only (open circles) or PBS and anti-Ly-6G (black circles) and *R*. *typhi*-infected mice that were treated with isotype (open squares) or anti-Ly-6G antibody (black squares); n = 9–10 for each group). The data show the mean ± SEM of the clinical score obtained from two independent experiments. Survival rates (y-axis) of anti-Ly-6G- and isotype antibody-treated mice were compared using the Log-rank Mantel Cox test (*p* = 0.7778; non-significant). Spleens of *R*. *typhi*-infected neutrophil-depleted mice were still strongly enlarged. The inserted photograph shows two spleens from non-infected control mice that received PBS (left) and three spleens from neutrophil-depleted *R*. *typhi*-infected mice (**B**). Bacterial burden was determined by *PrsA* qPCR (y-axis) in liver, spleen, brain and lung (x-axis) of *R*. *typhi*-infected isotype-treated (white bars) or anti-Ly-6G-treated CB17 SCID mice (black bars) at the time of death (n = 10). The mean ± SEM is depicted (**C**). The percentage of CD11b^+^GR1^hi^ neutrophils (left) and CD11b^+^GR1^low^ MΦ/monocytes (right) (y-axis) in the spleens from the very same mice were determined by flow cytometry. Each dot represents a single mouse. The mean ± SEM is depicted. Data were analyzed by one-way ANOVA followed by Tukey post-test (left) or Kruskal Wallis test followed by Dunn´s post-test (right) after D´Agostino and Pearson normality test. Asterisks indicate statistically significant differences (*p<0.05, **p<0.01, ***p<0.001) (**D**). GPT was determined in sera of infected CB17 SCID mice that received isotype (open squares) or anti-Ly-6G antibody (black squares) (n = 8–9) at the time of death. Control animals were treated with anti-Ly-6G but received PBS instead of *R*. *typhi* (black circles). Each dot represents a single mouse. Mean ± SEM from combined results of two independent experiments are shown. Data were analyzed by Kruskal-Wallis test followed by Dunn´s post-test after D´Agostino and Pearson normality test. Asterisks indicate statistically significant differences (**p<0.01). Photographs show a representative liver of an isotype-treated (left) and an anti-Ly6G-treated *R*. *typhi*-infected CB17 SCID mouse (right) (**E**). Representative HE stainings of histological sections of the liver of an isotype-treated and an anti-Ly-6G-treated *R*. *typhi*-infected CB17 SCID mouse at the time of death are shown. Black arrows point to necrotic lesions. Necrosis was not observed in anti-Ly-6G-treated mice. Open arrows point to cellular infiltrates (**F**).

Recent data show that neutrophils largely contribute to bacterial elimination in the liver [27–30]. Having shown that neutrophils infiltrate the liver of *R*. *typhi*-infected CB17 SCID mice, we further elucidated the contribution of neutrophils to bacterial elimination and protection against *R*. *typhi*. For this purpose neutrophils were depleted in *R*. *typhi*-infected CB17 SCID mice by the application of anti-Ly-6G antibody beginning at day 6 post infection before neutrophils start to rise (Fig 2B). Anti-Ly-6G treatment was repeated every 3 days. *R*. *typhi*-infected control mice received equal amounts of isotype antibody instead. Additional control groups of CB17 SCID mice were treated with either PBS or anti-Ly-6G but were non-infected. Success of neutrophil depletion was examined by flow cytometry of blood cells from infected CB17 SCID mice 1 day after the second antibody application. At this point in time (day 10 post infection), CD11b^+^Ly-6G^hi^ neutrophils represented approximately 65% of the CD11b^+^ cells in the blood of *R*. *typhi*-infected control mice and were completely absent in *R*. *typhi*-infected CB17 SCID mice that received anti-Ly-6G antibody (Fig 6A). However, neutrophil depletion did not alter the course of disease as monitored by clinical scoring (Fig 6B, left) and mice succumbed to the infection with similar kinetics as control mice (Fig 6B, right). Furthermore, neutrophil-depleted animals showed comparable bacterial burden in spleen, liver, brain and lung as *R*. *typhi*-infected control animals that were treated with isotype antibody (Fig 6C).

*R*. *typhi*-infected CB17 SCID mice still developed splenomegaly in the absence of neutrophils that was comparable to *R*. *typhi*-infected mice that were treated with isotype antibody (Fig 6B, insert). We further assessed the cellular composition of the spleen of all groups of mice including non-infected animals that received either PBS or anti-Ly-6G only. As expected, the CD11b^+^GR1^hi^ neutrophil population was absent in all mice that received anti-Ly-6G, whether infected or not (Fig 6D, left), while a reciprocally, although not significantly enhanced percentage, of CD11b^+^GR1^low^ MΦ/monocytes was observed in *R*. *typhi*-infected CB17 SCID mice in the absence of neutrophils. 53.5±3.8% of the spleen cells in neutrophil-depleted mice were CD11b^+^GR1^low^ MΦ/monocytes compared to 45.3±1.9% in control animals (Fig 6D, right), indicating that the absence of neutrophils is in part compensated by increased accumulation of MΦ/monocytes.

### Neutrophils are responsible for liver damage

Although neutrophils are known to be crucial for pathogen elimination in bacterial infections [31], an impact of these cells in bacterial control was not observed in *R*. *typhi*-infected CB17 SCID mice (Fig 6C). However, activated neutrophils can also mediate harmful reactions and can be involved in hepatic injury [32, 33]. We therefore analyzed liver pathology by measuring serum GPT and performing histological stainings. Indeed, neutrophil-depleted *R*. *typhi*-infected CB17 SCID mice were completely protected from liver damage. In the absence of neutrophils, *R*. *typhi*-infected CB17 SCID mice showed normal serum GPT levels. Livers from these mice appeared healthy and the gall bladders were clear (Fig 6E). Moreover, necrotic lesions were not detectable anymore in histological stainings although still many foci of infiltrating cells were visible (Fig 6F). These were positive for IBA1 and, thus, MΦ (S2B Fig). These results clearly show that neutrophils are solely responsible for liver necrosis in *R*. *typhi*-infected CB17 SCID mice.

### *R*. *typhi*-infected CB17 SCID mice show systemic inflammation which is unaltered in the absence of neutrophils

**Fig 7:**
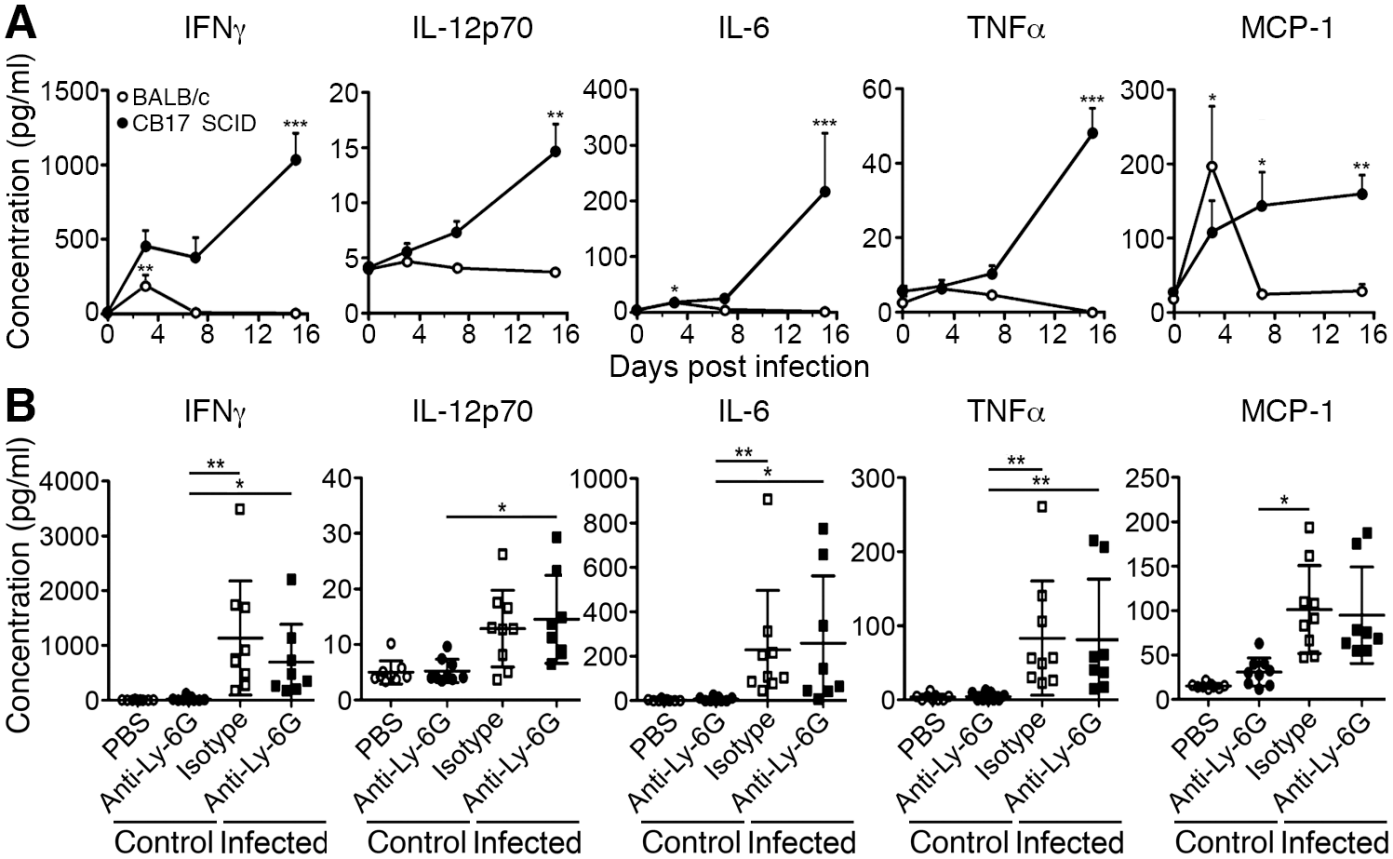
Neutrophil depletion does not alter systemic inflammatory response. Plasma cytokine levels (y-axis) were measured at indicated points in time (x-axis) in *R*. *typhi*-infected BALB/c (open symbols, n = 5–8) and CB17 SCID mice (black symbols, n = 5–8) by bead-based LEGENDplex assay. Data represent combined results from two independent experiments and are shown as mean ± SEM. Statistical significance was determined using Kruskall-Wallis test followed by Dunn´s post-test comparing samples from infected mice with samples from control mice (day 0) (**A**). Plasma cytokine levels (y-axis) from *R*. *typhi*-infected and PBS-treated CB17 SCID mice that either received isotype antibody or anti-Ly-6G as indicated on the x-axis were measured by bead-based LEGENDplex assay at the time of death. Combined results from two independent experiments are shown (n = 8–9). Each dot represents a single mouse. The mean ± SEM is shown. Statistical analysis was performed with Kruskall-Wallis test followed by Dunn´s post-test (**B**). Asterisks indicate statistically significant differences (*p<0.05, **p<0.01, ***p<0.001).

Although neutrophil-depleted *R*. *typhi*-infected CB17 SCID did not show liver damage anymore the animals succumbed to the infection. Thus, liver damage is not the cause of death which must have other reasons. One of these might be an overwhelming immune response. To gain insight into the immune response of CB17 SCID and wild-type BALB/c mice upon *R*. *typhi* infection we next analyzed cytokines in the blood. *R*. *typhi*-infected CB17 SCID mice showed steadily increasing release of inflammatory cytokines during the course of disease. This response was clearly dominated by IFNγ that reached plasma levels of 1034±180 pg/ml prior to death. In addition, slightly enhanced plasma levels of IL-6 (217±105 pg/ml), IL-12p70 (15±3 pg/ml), TNFα (48±7 pg/ml) and MCP-1 (160±25 pg/ml) were present (Fig 7A). GM-CSF was not significantly enhanced in *R*. *typhi*-infected CB17 SCID mice during the course of infection (S3 Fig). These results show that CB17 SCID mice mount a very strong systemic inflammatory response. In contrast, immunocompetent BALB/c wild-type mice produced significantly increased levels of IFNγ (191±70 pg/ml) in addition to MCP-1 (197±81 pg/ml) exclusively at day 3 post infection while IL-12p70, IL-6 and TNFα were not detectable at all in these mice (Fig 7A) and GM-CSF was not significantly elevated during the course of infection (S3 Fig).

We further clarified the impact of neutrophil-depletion on the inflammatory immune response in *R*. *typhi*-infected CB17 SCID mice. Serum levels of IFNγ, IL-12p70, IL-6, TNFα and MCP-1 were increased in all *R*. *typhi*-infected animals compared to control mice. Neutrophil-depletion, however, did not alter cytokine production compared to *R*. *typhi*-infected CB17 SCID mice that received isotype antibody (Fig 7B), demonstrating minor contribution of neutrophils to systemic inflammation.

### NK cells as well as MΦ produce IFNγ and MΦ represent the major source TNFα

**Fig 8:**
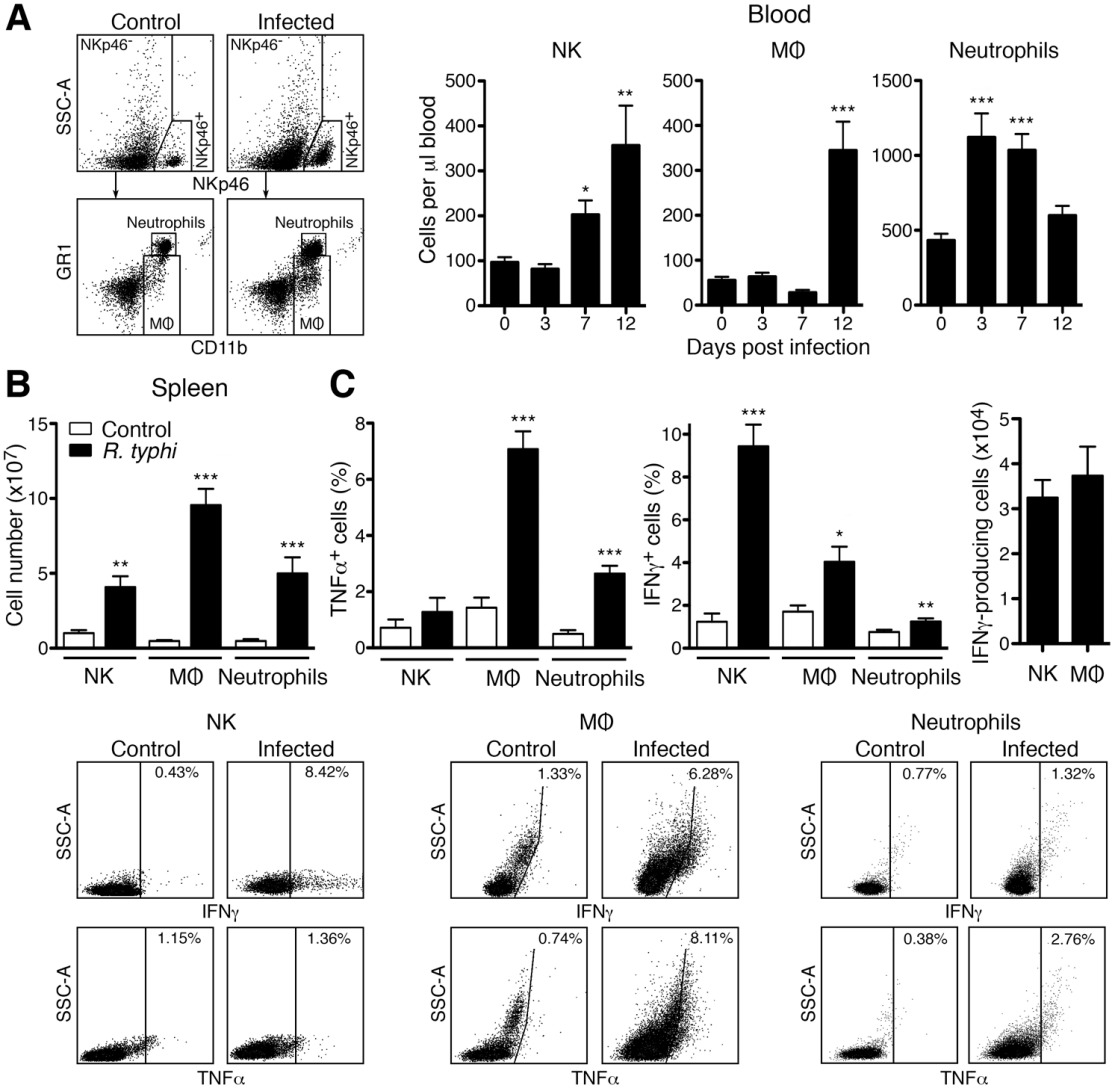
Expansion of NK cells, MΦ and neutrophils and cytokine expression by these cell populations. CB17 SCID mice (n = 8) were infected s.c. with 2×10^6^ sfu into the base of the tail while control animals received PBS (n = 8). Blood cells were analyzed by flow cytometric staining of NKp46, CD11b and GR1. Cells were gated on NKp46^+^ (NK cells) and NKp46^-^ cells that were further differentiated into MΦ (CD11b^+^GR1^low^) and neutrophils (CD11b^+^GR1^hi^) as indicated in the example dot plots on the left. Absolute numbers of NK cells, MΦ and neutrophils were determined per μl blood (y-axis) at indicated points in time (x-axis). Measurements from non-infected PBS-treated mice were used as "day 0" control. Statistical analysis was performed with Kruskall-Wallis test followed by Dunn´s post-test (**A**). 12h before the experiment was terminated (day 12) the animals received brefeldin A to prevent cytokine secretion by the cells *in vivo*. Spleen cells were stained for NKp46, CD11b, GR1, IFNγ and TNFα and analyzed by flow cytometry. Cells were gated as described above. Absolute cell counts (y-axis) of NK cells, MΦ and neutrophils as indicated on the x-axis were determined. Statistical analysis was performed with Mann-Whitney U test (two-tailed) (**B**). The percentage of IFNγ- and TNFα-expressing cells among NK cells, MΦ and neutrophils in the spleen of PBS-treated control mice (n = 7) and *R*. *typhi*-infected animals (n = 8) was analyzed. The dot plots below show example stainings of the indicated cell population. Graphs on the left and in the middle show the statistical analysis of the percentage of IFNγ-expressing and TNFα-expressing cells (y-axis) among each cell population (x-axis). The graph on the right shows the analysis of the absolute cell count of IFNγ-expressing cells (y-axis) among NK cells and MΦ (x-axis). Statistical analysis was performed with Mann-Whitney U test (two-tailed) (**C**). Asterisks indicate statistically significant differences (*p<0.05, **p<0.01, ***p<0.001).

Systemic inflammation in *R*. *typhi*-infected CB17 SCID mice was clearly dominated by IFNγ, a cytokine that is known to be predominantly produced by NK cells. Therefore, we further assessed the expansion of NK cells in addition to MΦ and neutrophils during the course of disease and cytokine expression by these cell populations. First, blood cells were stained for NKp46, CD11b and GR1 and absolute cell counts of NK cells (NKp46^+^), MΦ (CD11b^+^GR1^low^) and neutrophils (CD11b^+^GR1^hi^) after pregating on NKp46^-^ cells were determined. NK cells steadily increased during the course of infection and were enhanced approximately 3.5-fold on day 12 post infection in the blood (Fig 8A). Significantly enhanced numbers of MΦ were observed in the blood rather late in infection on day 12. The increase of these cells, however, was stronger (7-fold) compared to NK cells (3.5-fold) (Fig 8A). In contrast to NK cells and MΦ, neutrophils only transiently increased in the blood from day 3 to day 7 post infection and declined again until day 12 (Fig 8A) when the experiment was terminated.

We further analyzed cytokine expression by these cell populations and determined numbers of NK cells, MΦ and neutrophils in the spleen. For this purpose we prepreated the animals with brefeldin A 12 hours prior to the analysis to assess cytokine expression directly *ex vivo*. Spleen cells were then stained for NKp46, CD11b and GR1 to distinguish NKp46^+^ NK cells and CD11b^+^GR1^low^ MΦ and CD11b^+^GR1^hi^ neutrophils among NKp46^-^ as described above. In addition, cells were stained for intracellular IFNγ and TNFα. First, cell counts of NK cells, MΦ and neutrophils were determined. Numbers of both MΦ and neutrophils were strongly increased in the spleen of *R*. *typhi*-infected animals (MΦ: 9.56±1.09×10^6^; neutrophils: 5.00±1.08×10^6^) compared to control mice (MΦ: 0.48±0.13×10^6^; neutrophils: 0.47±0.07×10^6^). Thus, MΦ and neutrophil numbers were approximately 20-fold and 10-fold enhanced at this point in time (Fig 8B). Numbers of NK cells were generally higher compared to MΦ and neutrophils in CB17 SCID mice. 1.01±0.19×10^6^ NK cells were detectable in non-infected CB17 SCID control mice. NK cells significantly increased approximately 4-fold in the spleen of *R*. *typhi*-infected mice (4.08±0.72×10^6^) (Fig 8B). This increase corresponds to that observed in the blood (Fig 8A).

TNFα was predominantly detectable in MΦ. 7.08±0.64% of the MΦ expressed this cytokine. A lower proportion of neutrophils also expressed TNFα (2.65±0.28%) while TNFα expression by NK cells was negligible and not significantly enhanced (1.28±0.50%) (Fig 8C, left). However, 9.43±1.02% of the NK cells in the spleen expressed IFNγ. Surprisingly, IFNγ was also detectable in 4.04±1.02% of the MΦ while only 1.24±0.16% IFNγ-expressing cells were present among neutrophils (Fig 8C, middle). The absolute number of IFNγ-producing MΦ and NK cells was comparable in *R*. *typhi*-infected mice (Fig 8C). This is explained by the stronger increase of MΦ compared to NK cells (Fig 8B).

These results show that both NK cells and MΦ contribute to IFNγ production and that MΦ are a major source of TNFα in *R*. *typhi*-infected CB17 SCID mice.

### MΦ and neutrophils do not directly respond to *R*. *typhi in vitro*

**Fig 9:**
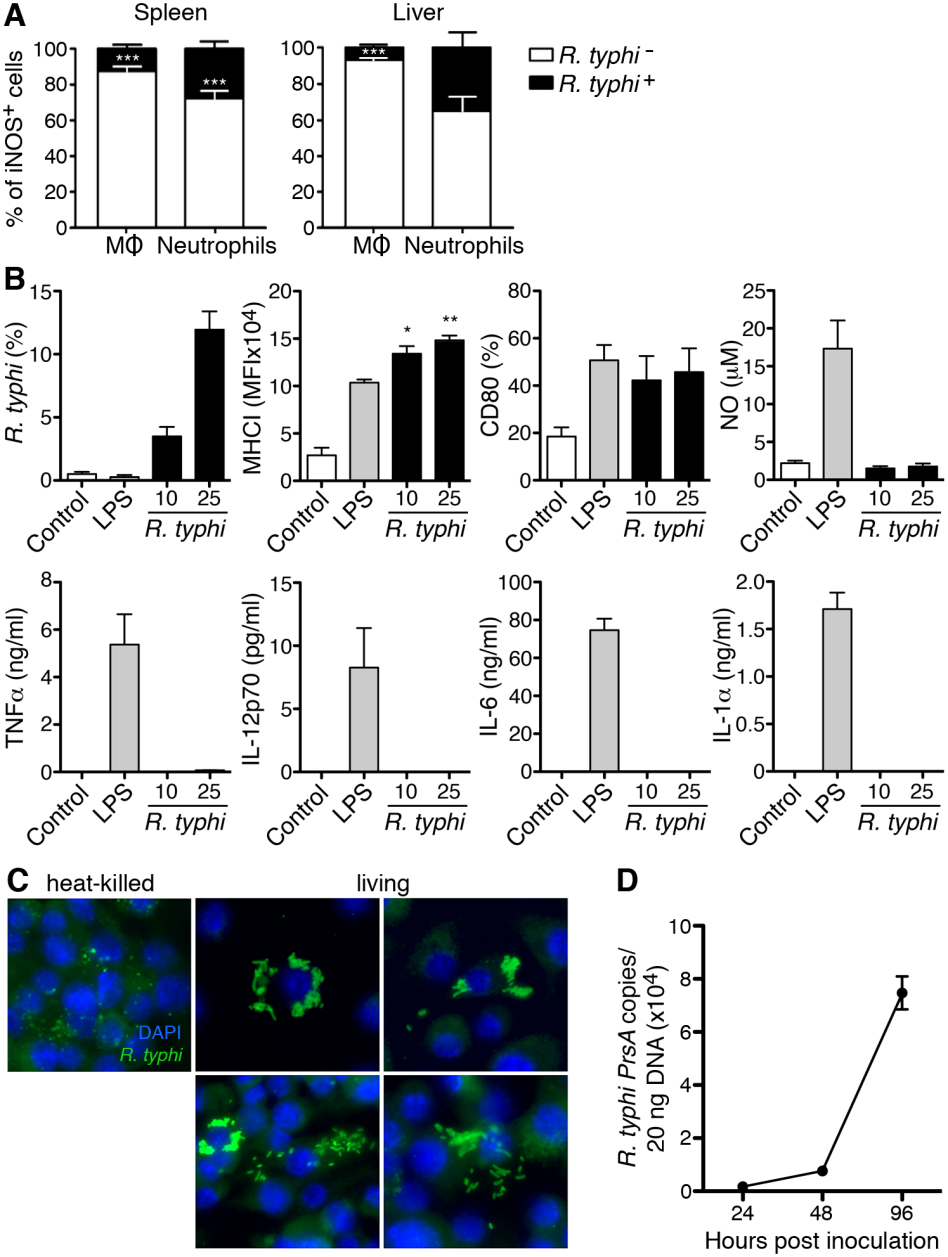
MΦ hardly respond to *R*. *typhi* and are uncapable to kill the bacteria. To analyze a correlation of bacterial uptake and activation status of CD11b^+^GR1^low^ MΦ /monocytes and CD11b^+^GR1^hi^ neutrophils *in vivo* the respective cell population in the spleen and liver of the mice described in Fig 5 was first gated on iNOS^+^ cells and then analyzed for bacterial content. The graphs show the percentage of *R*. *typhi*-positive cells (*R*. *typhi*^+^, black bars) and *R*. *typhi*-negative cells (*R*. *typhi-*, white bars) among the iNOS-expressing cells (y-axis) of the indicated cell population (x-axis). Statistical analysis was performed with Student´s T test (***p<0.001) (**A**). bmMΦ were infected with indicated amounts of purified bacteria per cell. Control cells were either left untreated (control) or stimulated with 100 ng/ml LPS (x-axis). After 24h cells were analyzed for the presence of *R*. *typhi* by flow cytometric staining. The percentage of *R*. *typhi*^+^ cells is shown (y-axis). MHCI and CD80 expression and the release of NO, TNFα, IL-12p70, IL-6 and IL-1 were analyzed after 48h (y-axis). Graphs show combined results from four independent experiments. Data were analyzed by one-way ANOVA followed by Tukey´s post-test. Asterisks indicate statistically significant differences compared to untreated cells (*p<0.05, **p<0.01) (**B**). bmMΦ were plated in chamber slides and either treated with heat-inactivated or living *R*. *typhi* as indicated. 10 copies per cell were used. After 48 h cells were stained for *R*. *typhi* with anti-*R*. *typhi* (BNI52) (green). Nuclei were stained with DAPI (blue). Images were taken at 800x magnification. One view of bmMΦ containing heat-inactivated, degrading bacteria and four views of bmMΦ containing living *R*. *typhi* are shown (**C**). Growth of *R*. *typhi* in bmMΦ that were infected with 5 copies per cell was analyzed. Bacterial content (y-axis) was quantified by *PrsA* qPCR at indicated points in time (x-axis). The graph shows combined results from three independent experiments (**D**).

As MΦ and neutrophils seem to be the dominant cell populations rising in *R*. *typhi*-infected CB17 SCID mice and show an activated phenotype, we further clarified if and how these cells react to *R*. *typhi*. In the experiment depicted in Fig 5, we first gated on iNOS^+^ cells among CD11b^+^GR1^low^ MΦ/monocytes and CD11b^+^GR1^hi^ neutrophils and analyzed the cells for *R*. *typhi* content. This analysis should demonstrate whether it is the *R*. *typhi*-harboring cells that express iNOS. Surprisingly, the vast majority of the iNOS-expressing CD11b^+^GR1^low^ MΦ/monocytes was *R*. *typhi*-negative (87.7±2.3% in the spleen and 92.8±1.3% in the liver (Fig 9A)) and the proportion of iNOS-expressing cells that contained bacteria was correspondingly small. Similar was also true for CD11b^+^GR1^hi^ neutrophils. Here, 72.3±4.1% of the iNOS-expressing cells in the spleen and 64.4±8.1% of those in the liver were negative for *R*. *typhi* (Fig 9A). Thus, activation of these cells does not correlate with bacterial phagocytosis.

Phagocytes such as MΦ usually recognize bacterial pathogens via pattern recognition receptors such as Toll-like receptors (TLR). These induce a classically activated phenotype including iNOS expression as observed in *R*. *typhi*-infected CB17 SCID mice, the release of inflammatory cytokines and the upregulation of costimulatory cell surface molecules [34]. The observation that MΦ and neutrophil activation did not correlate with bacterial uptake *in vivo* (Fig 9A), however, indicates that the cells may not directly react to the bacteria. To elucidate whether *R*. *typhi* activates MΦ, we generated bmMΦ from BALB/c mice and infected the cells with titrated amounts of *R*. *typhi in vitro*. Cells were analyzed for bacterial uptake and the expression of MHCI and CD80 by flow cytometry. In addition, NO, IL-6, IL-12 and TNFα were quantified in the supernatant by Griess reaction and LegendPlex assay. Stimulation with *E*. *coli* LPS was used as a control. After 24h approximately 12% of the cells that were infected with 25 *R*. *typhi* copies per cell were positive for *R*. *typhi* as determined by flow cytometry (Fig 9B). MΦ upregulated the expression of MHCI and CD80 48h after infection (Fig 9B). However, the cells neither released detectable amounts of inflammatory cytokines nor NO as it was observed upon stimulation with LPS (Fig 9B). These results show that MΦ do not react to *R*. *typhi* in a classical manner and further led to the question whether MΦ are capable to kill *R*. *typhi*. To clarify this issue we incubated bmMΦ with either living *R*. *typhi* or heat-killed *R*. *typhi* to be able to distinguish between living/replicating and degrading bacteria by immunofluorescence microscopy. As expected, fragments of degrading *R*. *typhi* particles were detectable after 48h in bmMΦ that received heat-killed bacteria (Fig 9C). In contrast, bmMΦ that were incubated with living *R*. *typhi* clearly contained intact and replicating bacterial particles at this point in time. In several cells proliferating bacteria clustered in rosette-like structures while other cells showed a more equal distribution of *R*. *typhi* in the cytosol. Interestingly, free *R*. *typhi* antigen not bound to particles was also observed in some cells in the cytosol (Fig 9C). Bacterial growth in bmMΦ *in vitro* was further confirmed by qPCR showing exponential increase of bacterial DNA in the cell culture within 96h (Fig 9D). Collectively these results show that *R*. *typhi* does not activate MΦ in a classical manner and that MΦ are incapable to kill the bacteria *in vitro*.

### *R*. *typhi*-infected BALB/c mice survive *R*. *typhi* infection and show weak liver pathology

**Fig 10:**
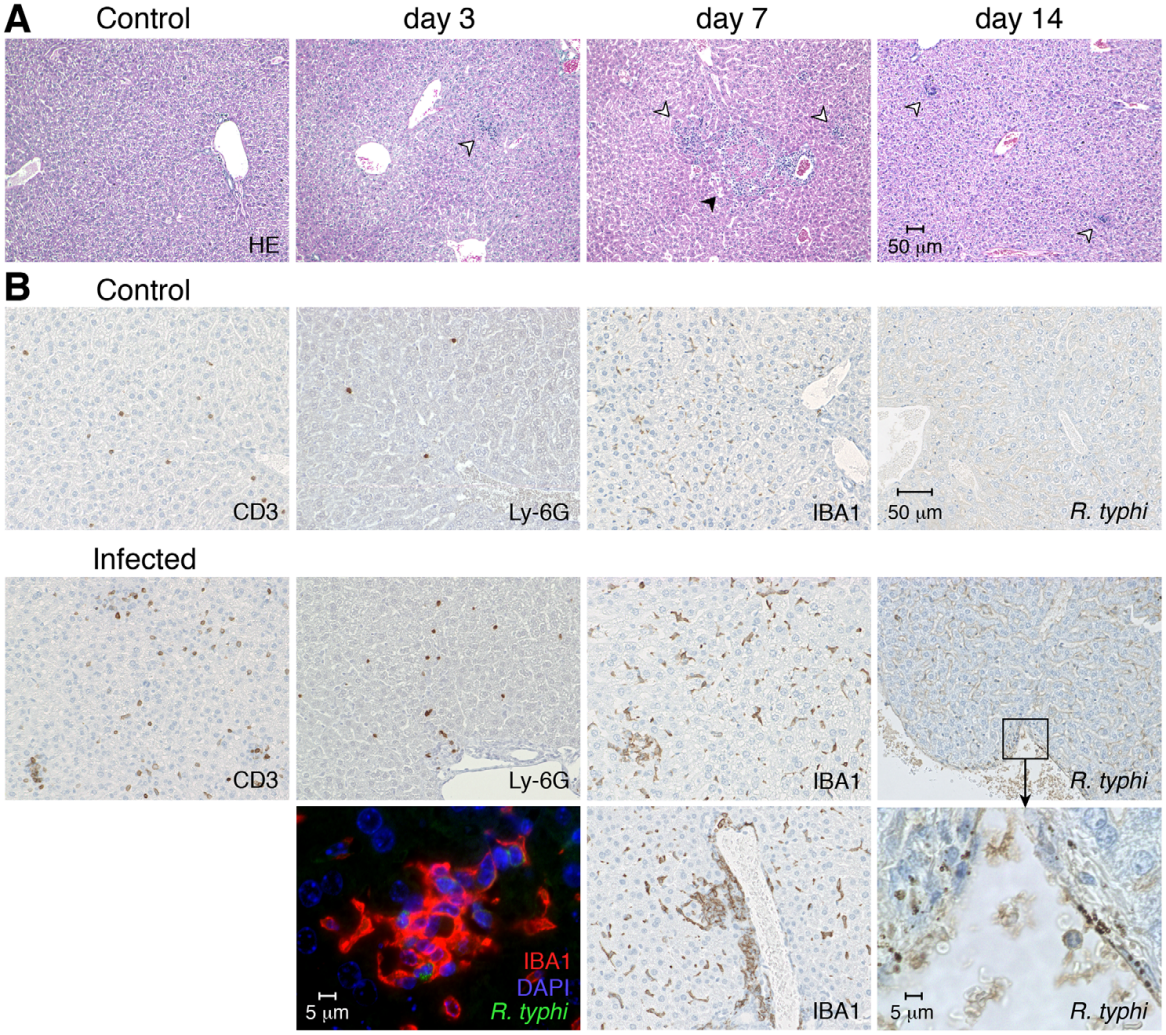
Temporary liver damage in BALB/c wild-type mice upon *R*. *typhi* infection. BALB/c mice were infected s.c. with 2×10^6^ sfu *R*. *typhi*. Liver sections were analyzed at indicated points in time post infection by staining with HE. Pictures show representative stainings of one out of five mice (**A**). Serial sections of the liver from PBS-treated BALB/c control mice or BALB/c mice infected s.c. with *R*. *typhi* as described above were stained for IBA1, Ly-6G, iNOS and *R*. *typhi* employing serum from a patient. For the staining of IBA1 two views are shown. In addition, co-staining of *R*. *typhi* (green), IBA1 (red) and DAPI (blue) was performed (**B**).

The results presented so far show that activated MΦ and neutrophils accumulate in immunodeficient CB17 SCID mice upon *R*. *typhi* infection and infiltrate the liver. Moreover, neutrophils revealed to be responsible for liver damage in these animals. To finally show whether these processes may also take place in immunocompetent mice, we again infected BALB/c wild-type mice. Although BALB/c wild-type mice do not show clinical symptoms of disease, histological analyses revealed that *R*. *typhi* infection affects the liver. Cellular infiltrates were visible in HE stainings of liver sections beginning on day 3 post infection, peaking on day 7 and declining until day 14 again (Fig 10A). At the peak of cellular infiltration on day 7, necrotic lesions were also visible in the livers of *R*. *typhi*-infected BALB/c mice (Fig 10A). Further stainings revealed that enhanced numbers of IBA1^+^ MΦ, Ly-6G^+^ granulocytes as well as T cells were detectable in the liver of *R*. *typhi*-infected animals compared to control BALB/c mice that were treated with PBS (Fig 10B). These cells were mainly found in the liver parenchyma. In addition, IBA1^+^ MΦ also accumulated around blood vessels (Fig 10B, below middle). The bacteria and/or bacterial antigen were detectable in endothelial cells (Fig 10B, below right) and in IBA^+^ MΦ (Fig 10B, below left) but not in the liver parenchyma. Liver damage in BALB/c wild-type mice, however, was generally mild. Necrotic lesions were small and rare and enhanced levels of serum GPT were not detectable (S3 Fig).

## Discussion

Immune competent BALB/c wild-type mice do not show symptoms of disease upon *R*. *typhi* infection, and the bacteria are quickly eliminated. Nonetheless, these mice show temporary liver damage. Liver dysfunction and hepatosplenomegaly are complications that frequently occur in patients with severe outcome of murine typhus [7]. In the current study we describe a murine model of *R*. *typhi* infection that reflects this pathology. T and B cell-deficient CB17 SCID mice succumb to the infection within approximately 3 weeks. The bacteria enter all analyzed organs, demonstrating systemic distribution. The most evident pathological changes, however, are splenomegaly and massive liver necrosis. The same observations were made in T and B cell-deficient BALB/c RAG2^-/-^ mice. These animals showed a comparable course and outcome of disease (liver necrosis, splenomegaly) as well as a similar bacterial distribution in the organs (S3 Fig). We therefore conclude that this outcome of disease is due to the lack of adaptive immunity in CB17 SCID mice rather than potential other effects of the mutation of the *Prkdc* gene that is responsible for the lack of T and B lymphocytes. Thus, CB17 SCID mice show a complete different outcome of disease than T and B cell-deficient C57BL/6 RAG1^-/-^ mice that do not show splenomegaly and liver pathology but develop brain inflammation months after *R*. *typhi* infection[21]. These observations suggest that the different genetic background that may influence the innate immune status of these mouse strains determines the outcome of disease.

Splenomegaly in CB17 SCID mice was mainly caused by the disproportionate accumulation of neutrophils and MΦ compared to other cells such as NK cells that showed comparatively moderate expansion. Transient increase of neutrophils in the blood and steady increase in the spleen may be a result of increasing exhaustion of the neutrophil reservoir in the bone marrow. Similar has been observed in high dose infection of mice with *Listeria* (*L*.) *monocytogenes* which led to neutrophil depletion in the bone marrow [35]. The factors that drive this massive MΦ and neutrophil expansion in *R*. *typhi*-infected CB17 SCID mice remain elusive. One important factor that is involved in myelopoiesis is the granulocyte/macrophage colony stimulating factor (GM-CSF). As a part of the emergency response to infection, GM-CSF induces the production and mobilization of granulocytes and MΦ from the bone marrow [36, 37]. GM-CSF together with macrophage colony stimulating factor (M-CSF) further promotes the maintenance, survival and functional activation of these cells at sites of injury [38–41]. GM-CSF, however, was not enhanced in the sera of *R*. *typhi*-infected CB17 SCID mice at any point in time (S3 Fig). CB17 SCID mice produced enhanced levels of IL-6, a cytokine that can directly stimulate granulopoiesis [42], upon *R*. *typhi* infection. IL-6 has been shown to be crucial for efficient neutrophil response against bacterial infections such as *L*. *monocytogenes* [43]. Thus, IL-6 may play a role in the generation and mobilization of neutrophils in *R*. *typhi*-infected CB17 SCID mice.

Enhanced numbers of both MΦ and neutrophils were detectable in the liver in several foci suggesting infiltration of these cells from the periphery. These cells were also found in association with necrotic lesion. In addition, large numbers of IBA1^+^ MΦ were equally distributed in the liver parenchyma. These cells may represent Kupffer cells, the resident MΦ of the liver, that are known to expand upon liver injury [44]. Thus, hyperplasia of Kupffer cells may also take place in *R*. *typhi*-infected CB17 SCID mice. Both MΦ and neutrophils ingested *R*. *typhi in vivo*. Infiltrating neutrophils constitute the first line of defense against most invading pathogens and are involved in the clearance of bacterial infections [31]. Upon systemic application the majority of pathogens is cleared in the liver early in the course of infection. Furthermore, recent data show that neutrophils rather than Kupffer cells account for bactericidal activity and bacterial elimination in the liver. This is evidenced by the fact that neutrophil-depleted mice show reduced capability to kill gram-positive as well as gram-negative bacteria in the liver [27–30]. For example, mice that were not able to mobilize neutrophils showed increased replication of the intracellular bacterium *L*. *monocytogenes* and died from the infection [45–47]. Here, neutrophils were essential in early defense against *L*. *monocytogenes* in the liver but not in the spleen or peritoneal cavity [48]. Neutrophil-depletion in *R*. *typhi*-infected CB17 SCID mice, however, did not result in increased bacterial load in the liver or other organs. This observation indicates a minor contribution of neutrophils to bacterial elimination and inefficient killing of ingested *R*. *typhi* although the cells exerted effector functions including the production of ROS, the expression of iNOS and subsequent NO release that are involved in bacterial killing [49]. These neutrophil effector functions are usually induced by the recognition of pathogen in addition to cytokines that are released during infection [50, 51]. Neutrophil activation, however, did not correlate with the uptake of *R*. *typhi*, indicating that neutrophils may not directly recognize the bacteria.

Endothelial cells are considered the main target cells of rickettsiae [5]. In concordance, *R*. *typhi* particles were detectable in endothelial cells in histological stainings of the livers from BALB/c wild-type and CB17 SCID mice. However, also hepatocytes may represent target cells. For example, *R*. *conorii* directly infects human hepatocytes inducing iNOS expression in these cells [52]. However, neither *R*. *typhi* particles nor *R*. *typhi* antigen were detectable in the liver parenchyma or in necrotic lesions in histological stainings of the liver from CB17 SCID mice but within infiltrating IBA1^+^ MΦ and Ly-6G^+^ granulocytes. Similar was also true for *C*. *burnetii* that was found in expanded MΦ in CB17 SCID mice and also causes hepatosplenomegaly [53]. Surprisingly, depletion of neutrophils did not alter bacterial load in the liver and other organs. Nonetheless, neutrophil-depletion completely prevented liver necrosis in *R*. *typhi*-infected CB17 SCID mice. Thus, liver damage in *R*. *typhi* infection is a result of immunopathological activity of neutrophils rather than direct hepatocyte damage by the bacteria themselves. In concordance with these observations neutrophil effector functions have been shown to exert cytotoxic effects and can cause severe hepatic injury [32, 33]. Furthermore, depletion of neutrophils as well as the application of antioxidants or protease inhibitors can prevent liver dysfunction in animal models of sepsis [54–56].

Depletion of neutrophils, however, did not protect *R*. *typhi*-infected CB17 SCID mice from death. This can be ascribed to overwhelming systemic inflammation that was unaltered in neutrophil-depleted animals. Thus, there is only minor contribution of neutrophils to this response that was characterized by the release of MCP-1 and inflammatory cytokines including IFNγ, TNFα, IL-6 and IL-12 that are important for defense against intracellular pathogens.

MΦ represent the major cellular source of TNFα, IL-6 and IL-12 [57–60] while MCP-1, a chemoattractant protein for monocytes, is produced by various types of cells including endothelial cells, MΦ and fibroblasts upon oxidative stress or exposure to cytokines [61]. In concordance, TNFα was mainly produced by MΦ in *R*. *typhi*-infected CB17 SCID mice. IL-6 and TNFα are critical for rapid response to tissue injury and infections and induce the production of acute phase reactants in the liver [62, 63] whereas IL-12 is the main inducer of IFNγ in NK cells and T cells [64, 65]. This cytokine assists in bacterial killing by activating MΦ bactericidal functions [66, 67]. Apart from NK cells we identified MΦ as a cellular source of IFNγ in *R*. *typhi*-infected CB17 SCID mice. MΦ have been demonstrated to be capable to produce this cytokine [68, 69] and intracellular pathogens such as *Mycobacterium tuberculosis* can directly induce its production in MΦ [70–72]. In addition, IFNγ production in MΦ is induced by IL-12 [73, 74] which was also enhanced in *R*. *typhi*-infected CB17 SCID mice. Interestingly, IL-12 and intracellular bacteria such as mycobacteria synergize in the induction of IFNγ in *in vitro* infected MΦ [72]. Finally, IFNγ induces its own expression in MΦ [75]. In this way, IL-12 and IFNγ can activate MΦ in an autocrine manner and further accelerate macrophage-driven inflammatory response. The observation that absolute cell counts of IFNγ-expressing NK cells and MΦ were equal strongly suggests that MΦ substantially contribute to IFNγ release in *R*. *typhi*-infected CB17 SCID mice. Expression of additional cytokines such as TNFα by MΦ further suggests that these cells play a dominant role in overall inflammation.

Upon bacterial infection MΦ usually get activated by the recognition of common bacterial components, so-called pathogen-associated molecular pattern (PAMP), that are detected by pattern recognition receptors (PRR) such as Toll-like receptors (TLR). TLR engagement generally leads to a classically activated phenotype of MΦ. This is characterized by the production of inflammatory cytokines including IL-6, IL-12 and TNFα, the expression of iNOS and subsequent release of NO [34, 76, 77] which is required for effective killing of intracellular bacteria such as mycobacteria [78–80]. In addition, classically TLR-activated MΦ upregulate cell surface molecules that are involved in antigen presentation including MHCI and II and costimulatory molecules such as CD80 and CD86 [81]. Indeed, high amounts of iNOS-expressing MΦ were detectable in *R*. *typhi*-infected CB17 SCID mice. As a gram-negative bacterium *R*. *typhi* possesses LPS [82, 83] and other PAMPs suggesting that *R*. *typhi* activates MΦ via TLR like TLR4. A role of TLR4 in the activation of innate immunity has been shown for example for *R*. *conorii*, a member of the SFG [84]. However, bmMΦ did not react to *R*. *typhi* with the production of NO and inflammatory cytokines but only upregulated MHCI and CD80 expression upon infection *in vitro*. Moreover, we observed that the majority of iNOS-expressing MΦ *in vivo* did not harbor *R*. *typhi*. These results strongly suggest that *R*. *typhi* does not activate MΦ in a classical manner via TLR. *R*. *typhi* probably enters these cells more or less unrecognized by PRR or may even specifically inhibit MΦ activation. Inhibition of MΦ activation by TG rickettsiae, however, has not been described in the literature. In concordance with the non-activated status of MΦ upon *R*. *typhi* infection *in vitro*, we further observed that the bacteria survive and replicate in bmMΦ *in vitro*. *R*. *typhi* has been shown before to infect murine as well as human MΦ [85, 86] which is also true for other rickettsiae such as *O*. *tsutsugamushi* [12, 13] and *R*. *akari* [87, 88]. The observation that MΦ are incapable to efficiently kill *R*. *typhi* suggests that these cells likely represent general target cells of *R*. *typhi* and maybe other rickettsial species. However, differences in MΦ activity may exist between susceptible and non-susceptible mouse strains. A comparative analysis of the bactericidal activity of MΦ from mice of different genetic background and different susceptibility to rickettsial infections has been described in one study. Here, neither peritoneal MΦ from susceptible C3H/HeN mice nor resistant BALB/c and C57BL/6 mice were capable to kill *R*. *akari in vitro* unless the cells were activated with lymphokines. If treated with lymphokines, MΦ from all of these strains were comparably competent in killing *R*. *akari* [88]. These findings argue against major differences in macrophage activity against rickettsiae although differences may exist with regard to different rickettsial species.

Our observations indicate that MΦ as well as neutrophils must become activated by other factors than recognition of the bacteria themselves via signaling receptors. As T cell-derived lymphokines are missing in C57BL/6 RAG1^-/-^ and CB17 SCID mice, we suggest that such activating signals may be released in the context of cellular damage that can be induced by *R*. *typhi* infection of various cell types in different organs and tissues in the beginning of disease. These signals may include endogenous danger signals such as heat shock proteins (HSP) and ATP that can be released into the environment under circumstances of cellular damage [89]. Endogenous danger signals can activate neutrophils [31, 90] and induce inflammatory responses including the release of IL-12 in MΦ [89, 91–93]. Cytokines like IL-12 and IFNγ may then further accelerate the inflammatory response as described above. Prolonged and deregulated release of mediators such as TNFα, IL-6 and NO has non-beneficial effects. For example, TNFα acts cytotoxic and both TNFα and IL-6 are involved in pathological conditions including septic shock and cachexia [63, 94] which is also true for NO [95].

Collectively we conclude that death of *R*. *typhi*-infected CB17 SCID mice is most likely due to overwhelming systemic inflammation driven by MΦ and other cells such as NK cells. Liver damage, however, is clearly an immunopathological effect mediated by neutrophil activity rather than direct destruction of hepatocytes by *R*. *typhi* itself. Since liver damage is also seen in *R*. *typhi*-infected BALB/c wild-type mice and in human *R*. *typhi* infection [7], this mechanism might also be operating in immune competent individuals. Here, an unbalanced immune state and impaired functionality of adaptive immunity, especially T cells, may foster these processes and this outcome of disease.

## Supporting Information

S1 Fig Development of liver damage in CB17 SCID mice. CB17 SCID mice were infected s.c. with 2×10^6^ sfu *R*. *typhi*. Liver sections were analyzed at indicated points in time post infection by staining with HE (upper panel). *R*. *typhi* was detected with patient serum (lower panel). Black arrows point to necrotic areas. Open arrows indicate cellular infiltrates that became visible beginning around day 7 post infection. At this point in time the bacteria were predominantly found in endothelial cells. Necrotic lesions were still absent. Infiltrating cells further increased until death around day 15. *R*. *typhi* was then detectable in foci of infiltrating cells that were either Ly-6G^+^ neutrophils or IBA^+^ MΦ as depicted in Fig 4.
(TIF)

S2 Fig Focal infiltration of IBA1^+^ MΦ in the liver of *R*. *typhi*-infected neutrophil-depleted CB17 SCID mice. Figures show an overview of a serial section of a liver from a neutrophil-depleted *R*. *typhi*-infected CB17 SCID mouse stained with HE and for IBA1. Images were taken at 2-fold magnification. Several foci of infiltrating IBA1^+^ MΦ are visible. Arrows point to some of the larger clusters of IBA1^+^ MΦ.
(TIF)

S3 Fig BALB/c RAG2^-/-^ mice show the same course of disease as CB17 SCID mice and BALB/c mice are asymptomatic upon *R*. *typhi*-infection. T and B cell-deficient BALB/c RAG2^-/-^ mice (n = 5; black symbols) were infected s.c. with 2×10^6^ sfu into the base of the tail while control animals received PBS (n = 5; open symbols). Survival and the clinical score of the animals during the course of disease is depicted. BALB/c RAG2^-/-^ mice succumbed to the infection within 17 days. The animals lost weight and developed a clinical score with similar kinetics as *R*. *typhi*-infected CB17 SCID mice (Fig 1 and S3C Fig). Comparable to CB17 SCID mice also BALB/c RAG2^-/-^ mice developed splenomegaly (upper insert) and dramatic liver necrosis (lower insert) (**A**). Spleen, brain, liver and lung of *R*. *typhi*-infected BALB/c RAG2^-/-^ mice were analyzed for bacterial content by qPCR at the time of death. Similar to CB17 SCID mice *R*. *typhi*-infected BALB/c RAG2^-/-^ mice showed highest bacterial burden in the spleen followed by the brain, lung and liver (**B**). BALB/c wild-type mice (n = 8) and CB17 SCID mice (n = 8) were infected s.c. with 2×10^6^ sfu into the base of the tail. Survival, clinical score, serum levels of GPT (n = 6–8 for each group) and GM-CSF in plasma (n = 5–8 for each group) (y-axis) were assessed at indicated points in time (x-axis). BALB/c wild-type mice did not show symptoms of disease at any point in time and all mice survived the infection. Significantly elevated levels of GPT were not observed in BALB/c wild-type mice. Significantly enhanced levels of GM-CSF were neither produced by CB17 SCID nor BALB/c wild-type during the course of infection (**C**).
(TIF)
