# Supplementary figures and images for: Liver Necrosis and Lethal Systemic Inflammation in a Murine Model of Rickettsia typhi Infection: Role of Neutrophils, Macrophages and NK Cells
Source: PLoS Negl Trop Dis. 2016 Aug 22;10(8):e0004935. doi: 10.1371/journal.pntd.0004935 (PMC4993389; doi:10.1371/journal.pntd.0004935)

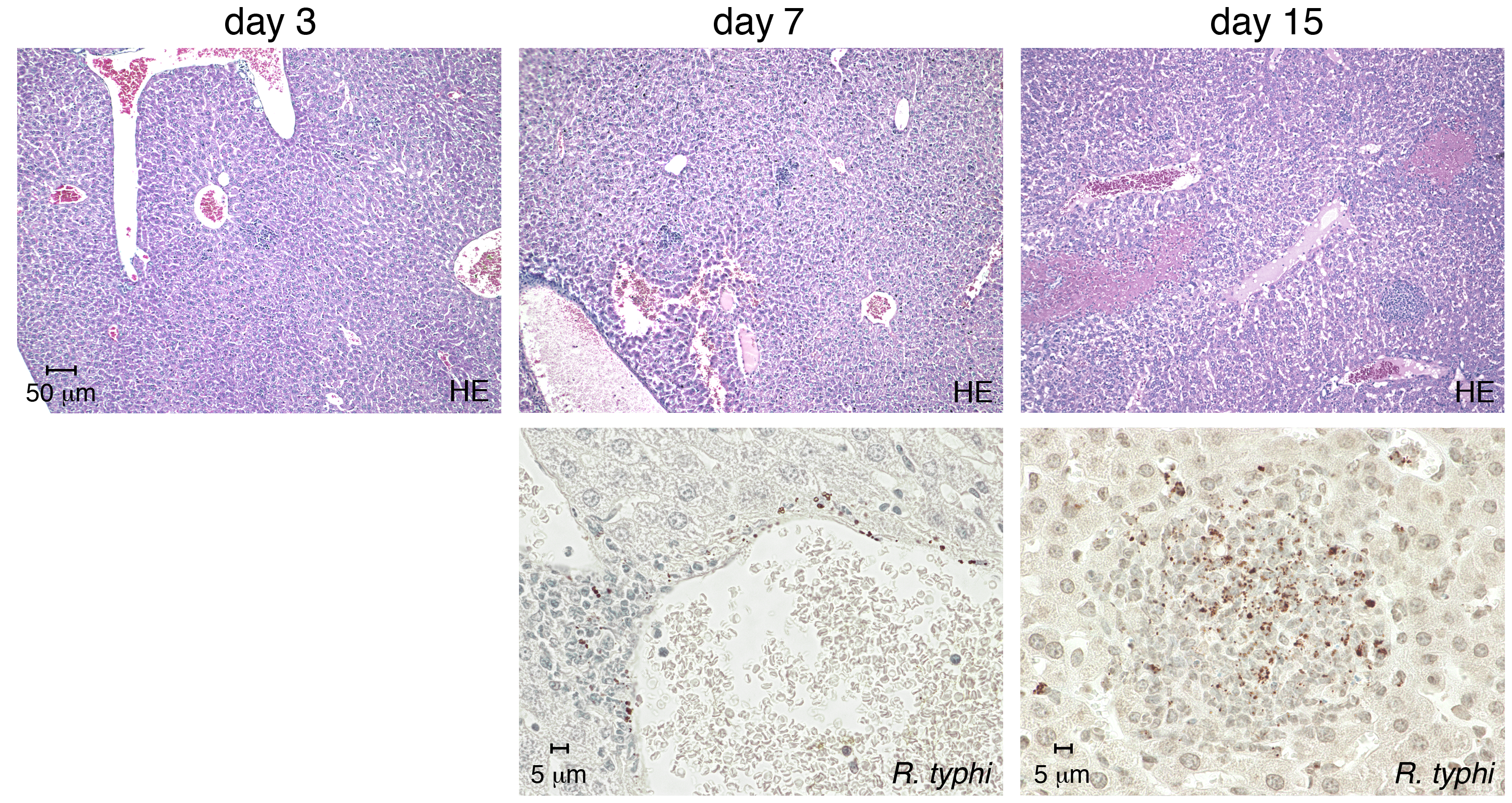

Supplement: S1 Fig — CB17 SCID mice were infected s.c. with 2×10^6^ sfu R. typhi. Liver sections were analyzed at indicated points in time post infection by staining with HE (upper panel). R. typhi was detected with patient serum (lower panel). Black arrows point to necrotic areas. Open arrows indicate cellular infiltrates that became visible beginning around day 7 post infection. At this point in time the bacteria were predominantly found in endothelial cells. Necrotic lesions were still absent. Infiltrating cells further increased until death around day 15. R. typhi was then detectable in foci of infiltrating cells that were either Ly-6G+ neutrophils or IBA+ MΦ as depicted in Fig 4. (TIF) [file pntd.0004935.s001.tif]

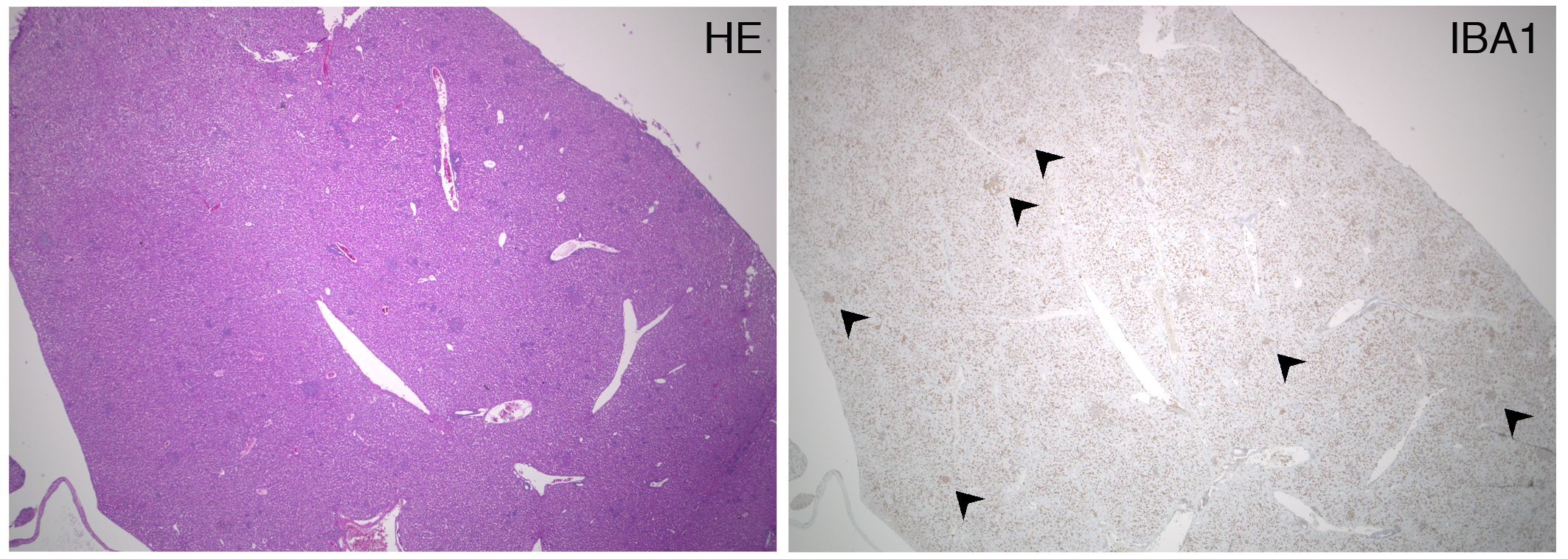

Supplement: S2 Fig — Figures show an overview of a serial section of a liver from a neutrophil-depleted R. typhi-infected CB17 SCID mouse stained with HE and for IBA1. Images were taken at 2-fold magnification. Several foci of infiltrating IBA1+ MΦ are visible. Arrows point to some of the larger clusters of IBA1+ MΦ. (TIF) [file pntd.0004935.s002.tif]

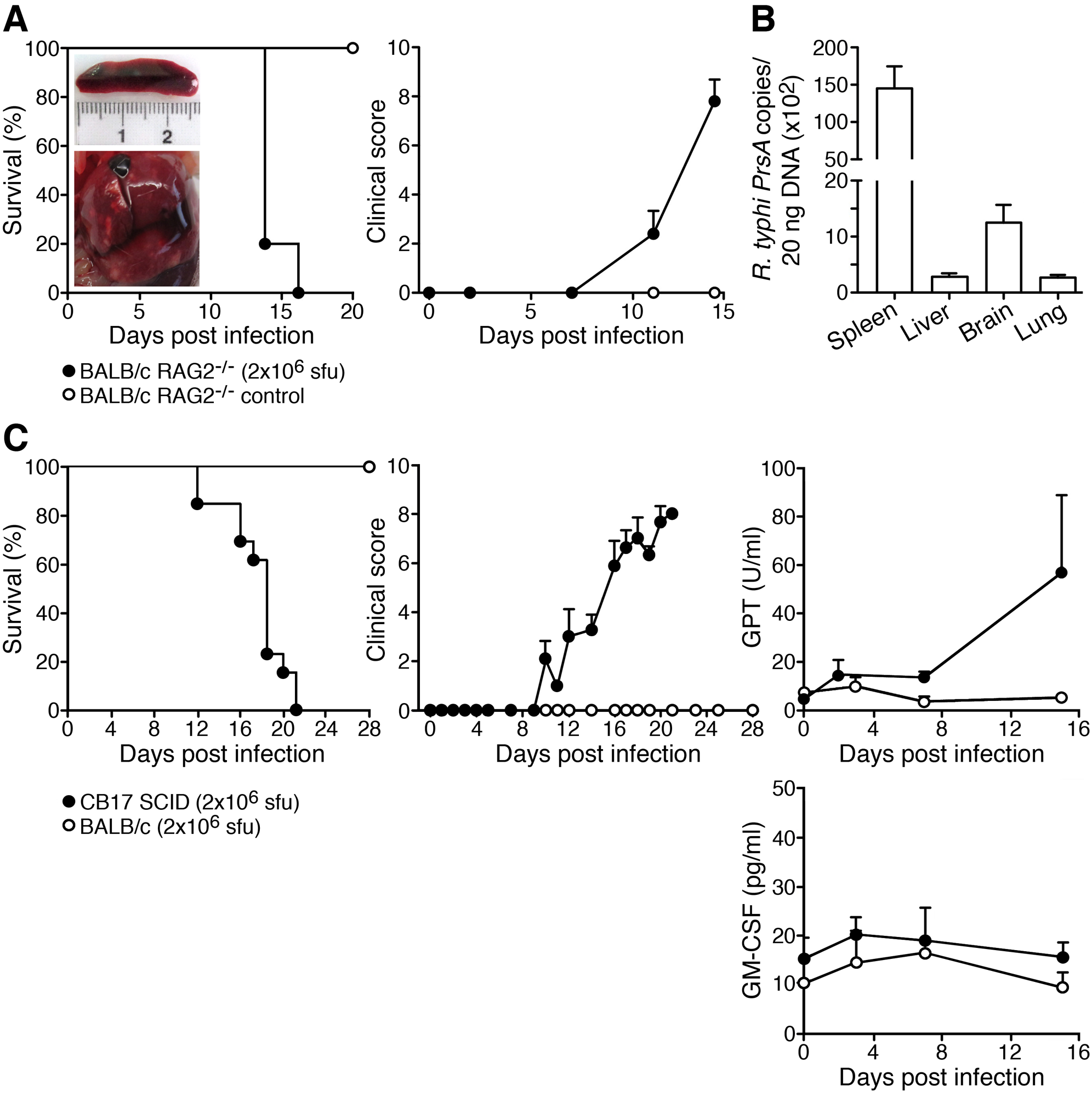

Supplement: S3 Fig — T and B cell-deficient BALB/c RAG2-/- mice (n = 5; black symbols) were infected s.c. with 2×10^6^ sfu into the base of the tail while control animals received PBS (n = 5; open symbols). Survival and the clinical score of the animals during the course of disease is depicted. BALB/c RAG2-/- mice succumbed to the infection within 17 days. The animals lost weight and developed a clinical score with similar kinetics as R. typhi-infected CB17 SCID mice (Fig 1 and S3C Fig). Comparable to CB17 SCID mice also BALB/c RAG2-/- mice developed splenomegaly (upper insert) and dramatic liver necrosis (lower insert) (A). Spleen, brain, liver and lung of R. typhi-infected BALB/c RAG2-/- mice were analyzed for bacterial content by qPCR at the time of death. Similar to CB17 SCID mice R. typhi-infected BALB/c RAG2-/- mice showed highest bacterial burden in the spleen followed by the brain, lung and liver (B). BALB/c wild-type mice (n = 8) and CB17 SCID mice (n = 8) were infected s.c. with 2×10^6^ sfu into the base of the tail. Survival, clinical score, serum levels of GPT (n = 6–8 for each group) and GM-CSF in plasma (n = 5–8 for each group) (y-axis) were assessed at indicated points in time (x-axis). BALB/c wild-type mice did not show symptoms of disease at any point in time and all mice survived the infection. Significantly elevated levels of GPT were not observed in BALB/c wild-type mice. Significantly enhanced levels of GM-CSF were neither produced by CB17 SCID nor BALB/c wild-type during the course of infection (C). (TIF) [file pntd.0004935.s003.tif]
